# Microarray analyses reveal genes related to progression and prognosis of esophageal squamous cell carcinoma

**DOI:** 10.18632/oncotarget.20232

**Published:** 2017-08-12

**Authors:** Mao Qixing, Dong Gaochao, Xia Wenjie, Wang Anpeng, Chen Bing, Ma Weidong, Xu Lin, Jiang Feng

**Affiliations:** ^1^ Department of Thoracic Surgery, Jiangsu Cancer Hospital, Jiangsu Institute of Cancer Research, Nanjing Medical University Affiliated Cancer Hospital, Nanjing, China; ^2^ The Fourth Clinical College of Nanjing Medical University, Nanjing, China; ^3^ Jiangsu Key Laboratory of Molecular and Translational Cancer Research, Nanjing Medical University Affiliated Cancer Hospital, Cancer Institute of Jiangsu Province, Nanjing, China

**Keywords:** esophageal squamous cell carcinoma, biomarker, progression, oncomine, prognosis

## Abstract

Esophageal squamous cell carcinoma is a high morbidity and mortality cancer in China. Here are few biomarkers and therapeutic targets. Our study was aimed to identify candidate genes correlated to ESCC. Oncomine, The Cancer Genome Atlas, Gene Expression Omnibus were retrieved for eligible ESCC data. Deregulated genes were identified by meta-analysis and validated by an independent dataset. Survival analyses and bioinformatics analyses were used to explore potential mechanisms. Copy number variant analyses identified upstream mechanisms of candidate genes. In our study, top 200 up/down-regulated genes were identified across two microarrays. A total of 139 different expression genes were validated in GSE53625. Survival analysis found that nine genes were closely related to prognosis. Furthermore, Gene Ontology analyses and Kyoto Encyclopedia of Genes and Genomes analyses showed that different expression genes were mainly enriched in cell division, cell cycle and cell-cell adhesion pathways. Copy number variant analyses indicated that overexpression of *ECT2* and other five genes were correlated with copy number amplification. The current study demonstrated that *ECT2* and other eight candidate genes were correlated to progression and prognosis of esophageal squamous cell carcinoma, which might provide novel insights to the mechanisms.

## INTRODUCTION

Esophageal squamous cell carcinoma (ESCC), one main subtype of esophageal cancer, accounts for more than 90% of esophageal cancer cases in China [1, 2]. Based on the National Central Cancer Registry of China (NCCR), esophageal cancer is 4th lethal cancer in China [3]. Over the past decades major progress has been made in diagnosis and treatment, the morbidity and mortality of ESCC present declining tendency [2, 4]. However, 5-year survival of ESCC is still less than 25%, which is dismal [5]. Thus, further understanding the potential biological mechanisms and exploring novel biomarkers are urgently needed.

Microarray technology provides a high-throughput and powerful tool to create large amounts of data including mRNA expression, DNA methylation, and microRNA expression [6, 7]. Published studies have identified many novel tumor biomarkers and potential therapeutic targets by microarray data [8–10]. The Cancer Genome Atlas (TCGA) and Gene Expression Omnibus (GEO) are two common public platforms archiving these data [11]. The wide range of microarrays in public archives provided an opportunity to reuse several microarrays to identify several novel targets [7, 12]. Oncomine currently includes gene expression and sample data from 500 cancer types [13]. There are more than 490 datasets and nearly 40,000 measured samples [14]. The data are normalized and analyzed using standard protocols. Based on the large amount data, Oncomine provides several analysis tools including differential expression analysis, co-expression analysis and comparing analysis [15]. For example, Clermont reported oncogenic role for CBX2 in pan-cancer [16]. Barfeld’s investigation revealed a novel signature for prostate cancer [17]. However, to our knowledge, few such studies have been conducted in ESCC. To fill in this gap, we conducted a study using Oncomine analysis tool to identify novel differential expression genes and potential pathways, which might provide new therapeutic targets for ESCC.

## RESULTS

### **Meta** analysis of significantly deregulated genes in ESCC

**Figure 1:**
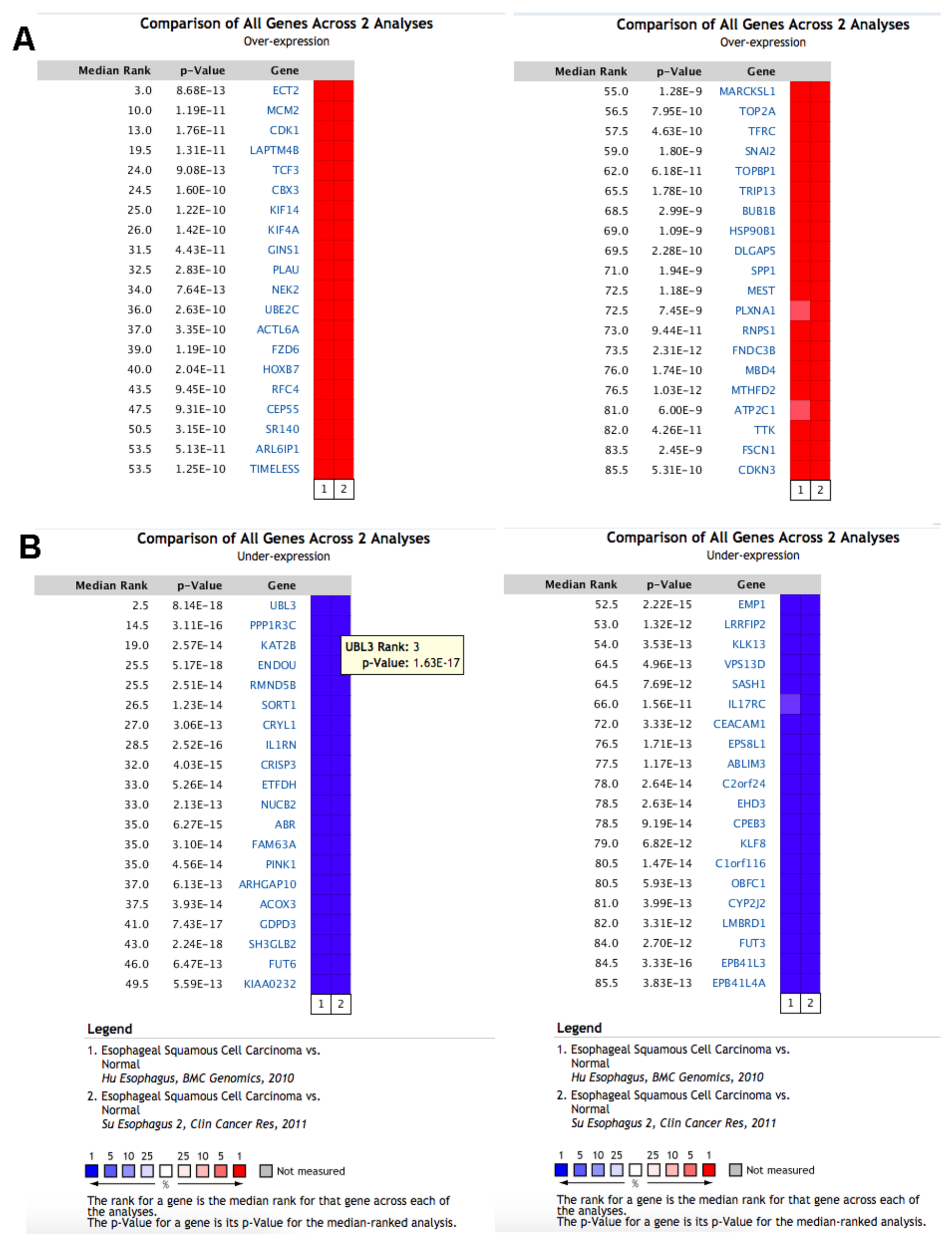
The top 80 genes that were significantly deregulated in ESCC across two independent microarrays retrieved from the Oncomine database **(A)** The top 40 genes were significantly up regulated. **(B)** The top 40 genes were significantly down regulated. The two microarrays were Su’s ESCC Statistics (52 ESCC tissues and 53 normal tissues) and Hu’s ESCC Statistics (17 ESCC tissues and 17 normal tissues). The genes labeled in red and in blue represent up/down-regulated in each microarray.

**Figure 2:**
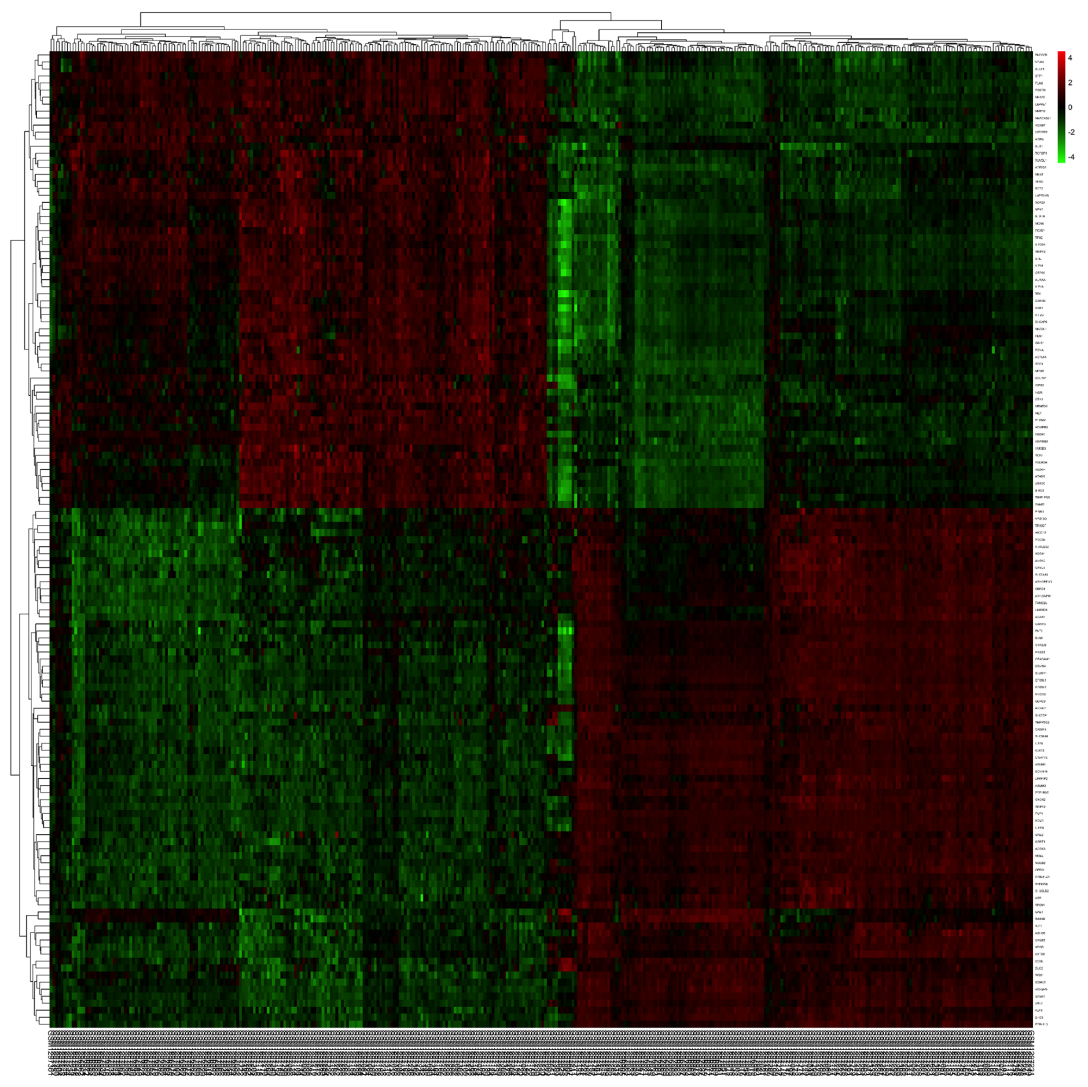
The heatmap revealed the overlapped differentially expressed genes between tumor and normal samples

**Figure 3:**
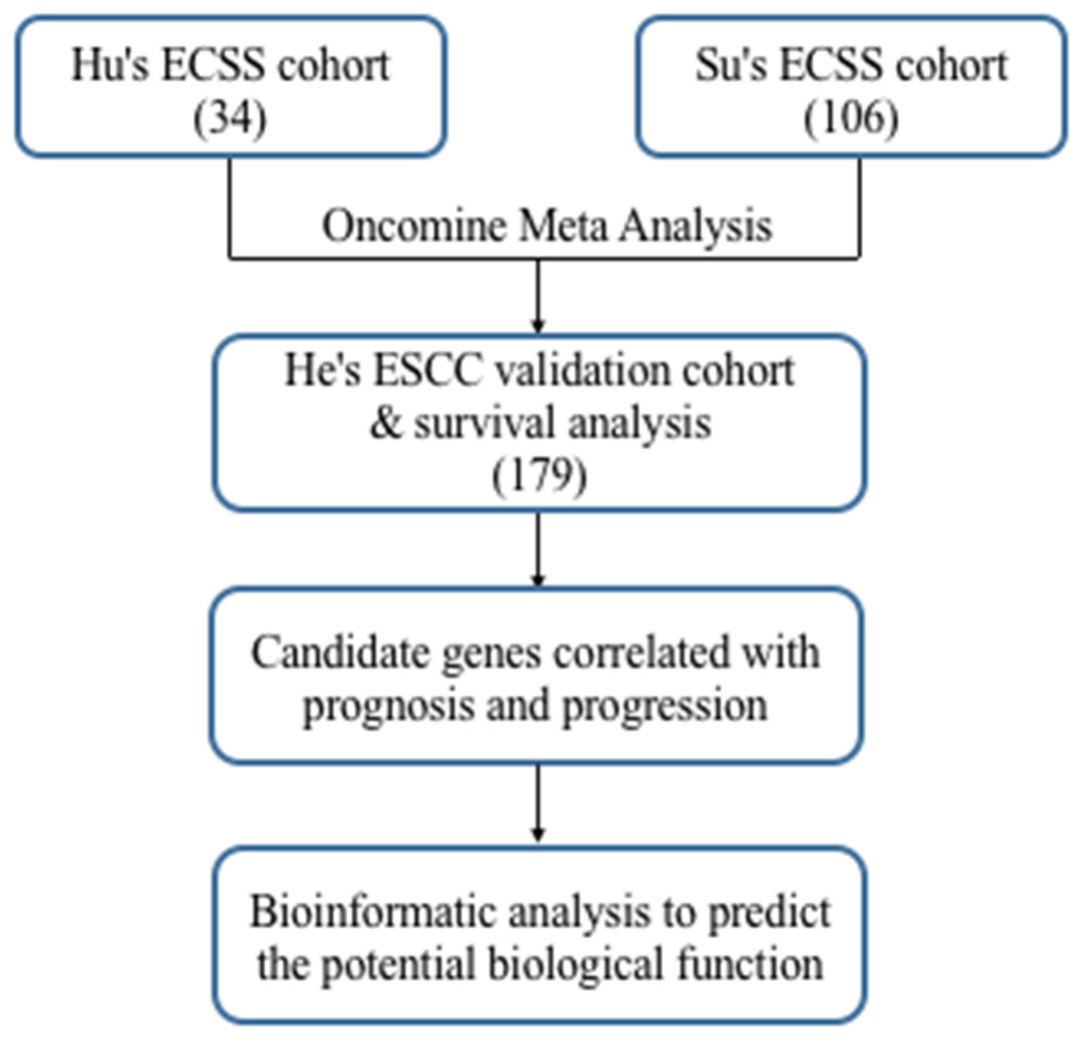
Flowchart for comprehensive analysis of the expression profiles and identification of the candidate genes correlated with progression and prognosis

There were two eligible independent ESCC microarray datasets in oncomine, which were Hu’s ESCC cohort with 34 cases and Su’s ESCC cohort covering 106 cases [18, 19]. A meta-analysis was performed across two microarray datasets. Two microarray datasets showed highly consistent trend of mRNA expression in top ranked genes (Figure 1). We selected 200 of the top ranked genes with the smallest P-values from the in over/under expression patterns (p<7.23E-07 and p<8.04E-12, fold change>2, respectively) (Supplementary Tables 1&2). In addition, different expression genes with fold change>2 were identified from GSE53625 dataset for validation. Overlap between results of Oncomine with outcome of GSE53625 showed that 139 genes were consistently and stably expression in ESCC (Figure 2). The flow chart of this study was showed in Figure 3.

### Candidate genes correlated with survival

**Figure 4:**
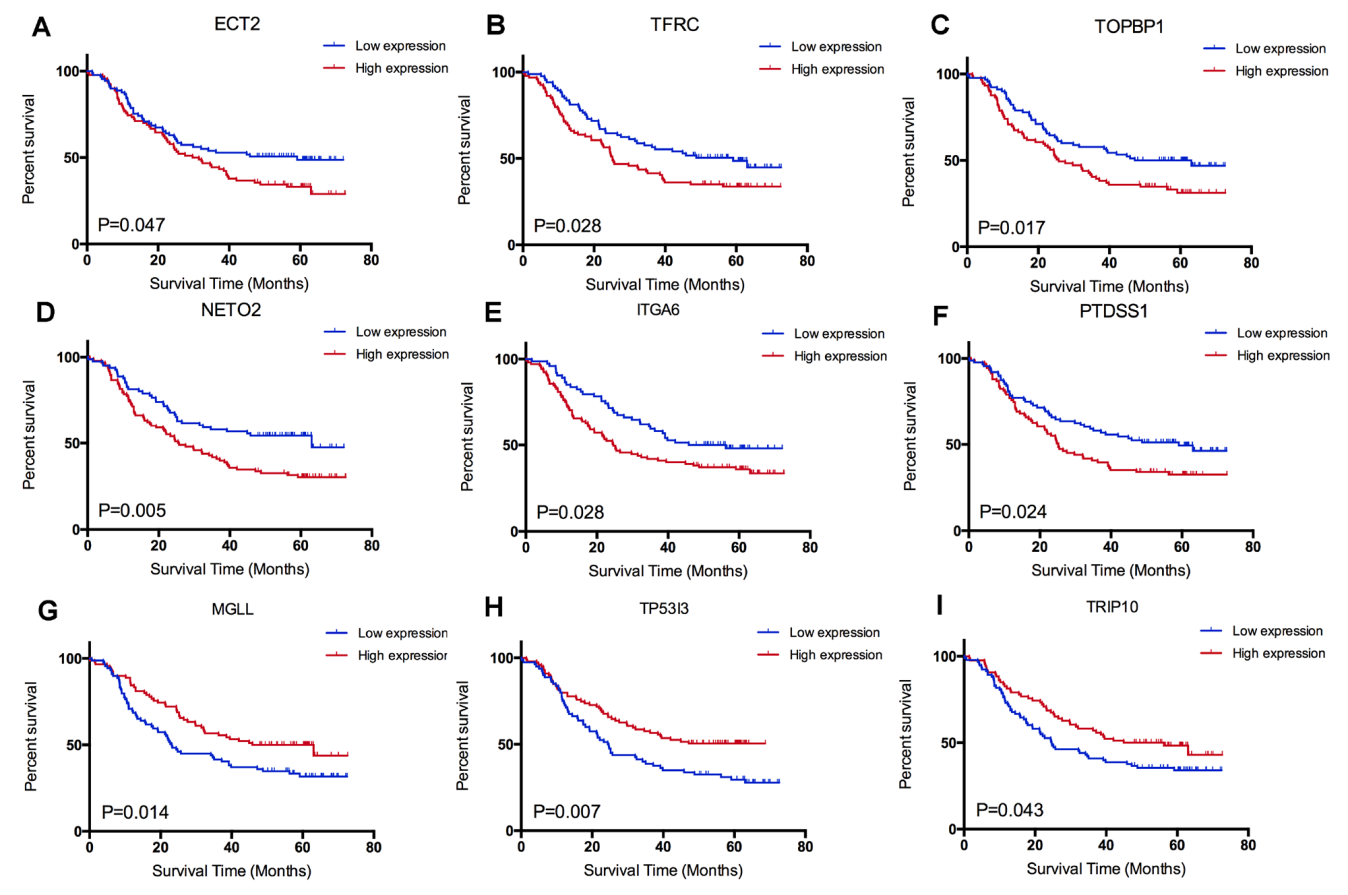
Survival analysis of candidate genes: **(A)** *ECT2*, **(B)** *TFRC*, **(C)** *TOPBP1*, **(D)** *NEOT2*, **(F)** *PTDSS1*, **(E)** *ITGA6*, **(G)** *MGLL*, **(H)** *TP53I3* and **(I)** *TRIP10*.

Combining with survival information of GSE53625, we next accessed the prognostic effect of candidate genes in ESCC. Log rank test indicated that nine genes were significant correlated with survival. Among them, we observed that up regulated of six genes (*ECT2, TFRC, TOPBP1, NETO2, PTDSS1, ITGA6*) were correlated with poor survival, while down regulated of three other genes (*MGLL, TP53I3, TRIP10*) were correlated with shorter survival (Figure 4). A comprehension literature search showed that *ECT2, TFRC* and *ITGA6* were reported to be prognostic biomarkers by previous ESCC investigations. Additionally, knockdown of *ECT2* and *ITGA6* expression suppressed esophageal cancer cell growth and proliferation (Table 1). However, few investigations reported the other six genes in carcinogenesis and development of ESCC.

**Table 1:** Fold changes and correlations between ESCC and nine candidate genes

Gene	Microarray data			Correlations with ESCC
	Up/down-regulated	Su’s ESCC	Hu’s ESCC	
ECT2	Up	4.346	5.87	Cancer progression and poor prognosis
TFRC	Up	2.544	3.652	Prognostic biomarker
TOPBP1	Up	2.34	2.11	-
NETO2	Up	2.689	2.566	-
PTDSS1	Up	2.083	2.214	-
ITGA6	Up	2.738	3.032	Proliferation of ESCC
MGLL	Down	-9.531	-3.109	-
TP53I3	Down	-6.323	-2.676	-
TRIP10	Down	-4.285	-2.408	-

The interrelationship between these candidate genes was further explored by GeneMANIA tool. GeneMANIA tool provided with network relationship of candidate genes by co-expression, shared protein domains and co-localization relationships based on published papers. The network, constructed of 27 nodes and 133 edges, showed a tightly correlation between candidate genes with other 18 key functional molecules. The network indicated potential mechanisms of ESCC (Supplementary Figure 1 & Supplementary Table 3).

### Potential roles of candidate genes

**Figure 5:**
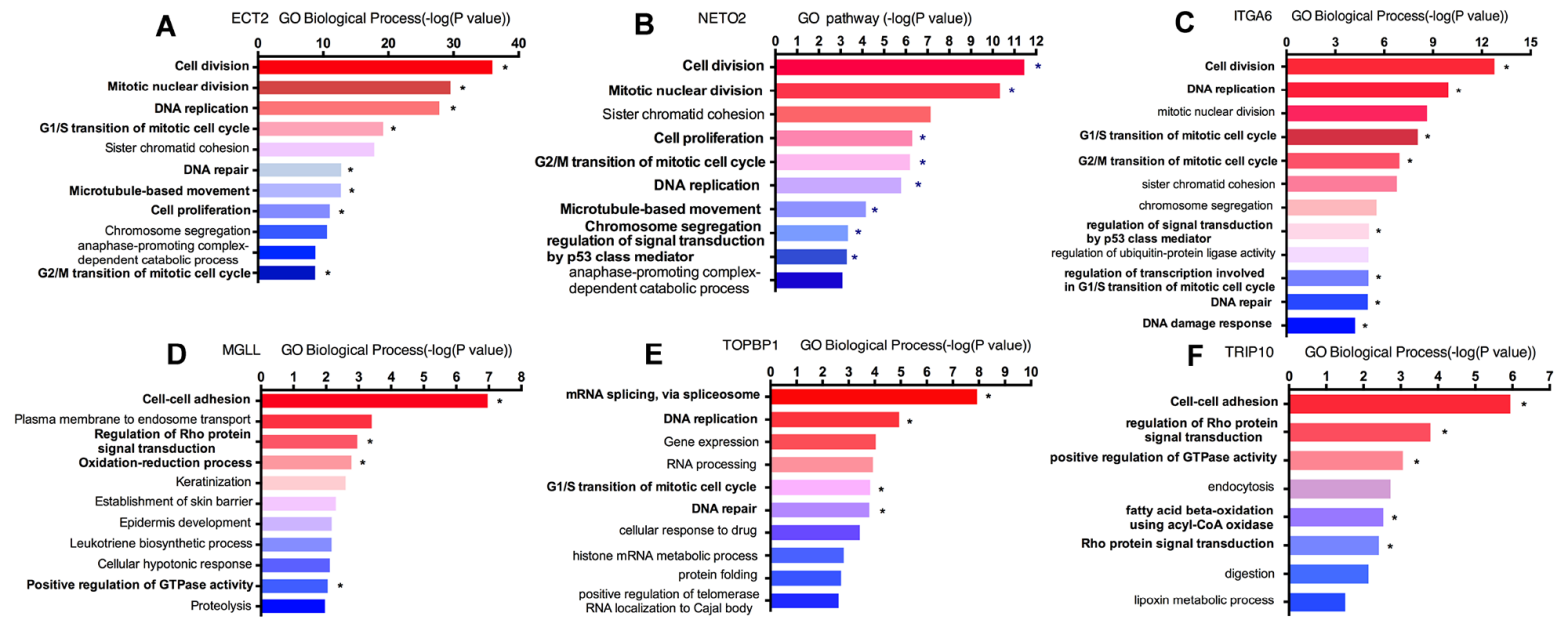
Enrichment analyses of candidate genes in Gene Ontology pathway “*” represented significant carcinogenic pathway. **(A)** ECT2 GO biological pathway analysis; **(B)** NETO2 GO biological pathway analysis; **(C)** ITGA6 GO biological pathway analysis; **(D)** MGLL GO biological pathway analysis; **(E)** TOPBP1 GO biological pathway analysis; **(F)** TRIP10 GO biological pathway analysis.

**Figure 6:**
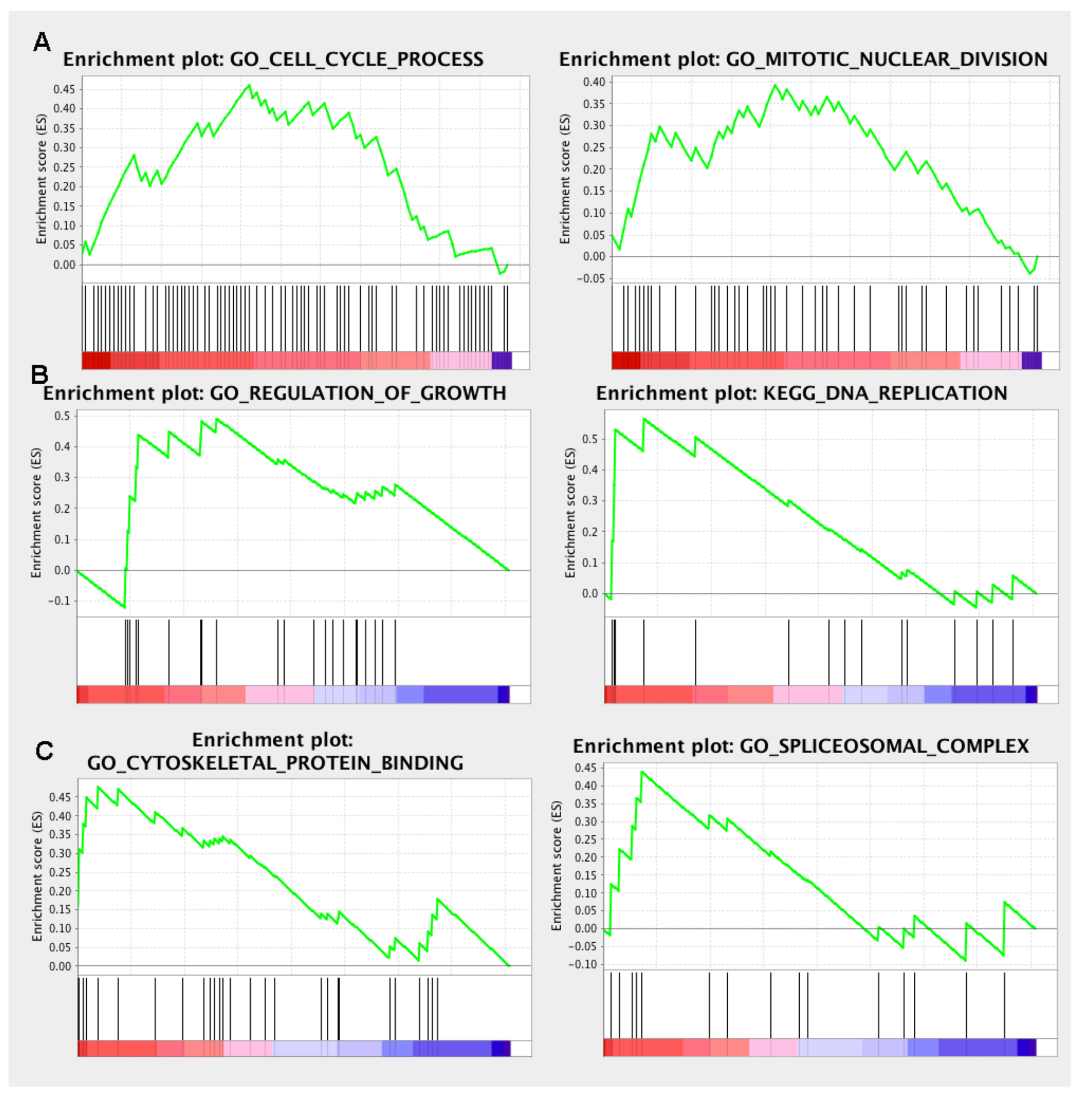
GSEA enrichment analysis of the co-expressed genes **(A)** ECT2 GSEA enrichment analysis; **(B)** TOPBP1 GSEA enrichment analysis; **(C)** TFRC GSEA enrichment analysis.

In order to explore the unique roles of each candidate genes, we performed Gene Ontology (GO) and KyotoEncyclopedia of Genes and Genomes (KEGG) pathway analysis by DAVID tool. We found that cell division, mitotic nuclear division, DNA replication and G1/S transition of mitotic cell cycle were enriched by a high p-value in *ECT2* analysis, implying a potential role of *ECT2* in cell division and cell cycles (Figure 5A). Additionally, we observed that *NETO2, ITGA6* and *TOPBP1* shared the same critical pathways like cell division, DNA repaired, cell cycles and cell proliferation (Figure 5B, 5C&5E). It indicated that *ECT2, NETO2, ITGA6* and *TOPBP1* might function as oncogenes to promote the progression of ESCC by regulating these pathways. Notably, *NETO2* and *ITGA6* also took part in the p53 class mediator signal transduction that was a critical carcinogenic pathway. GO analysis unveiled that *MGLL* and *TRIP10* participated in cell-cell adhesion, regulation of Rho protein signal transduction and positive regulation of GTPase activity, indicating that *MGLL* and *TRIP10* might regulate the invasion and adhesion ability of ESCC (Figure 5D&5F). Moreover, *TRIP10* was significantly correlated with fatty acid beta-oxidation using acyl-CoA oxidase pathway, while *MGLL* play a role in oxidation-reduction process, which were significantly correlated with carcinogenesis of ESCC. To investigate the potential altered pathways in the samples, Gene Set Enrichment Analysis (GSEA) was implemented between live/death groups. According to the result of *ECT2,* genes were mainly enriched in cell cycle process and mitotic nuclear division (Figure 6A). Moreover, we noticed that regulation of growth and DNA replication pathways were significantly enriched in *TOPBP1* (Figure 6B). As to *TFRC*, co-expression genes were enriched in cytoskeletal protein binding and spliceosomal complex pathways (Figure 6C).

**Figure 7:**
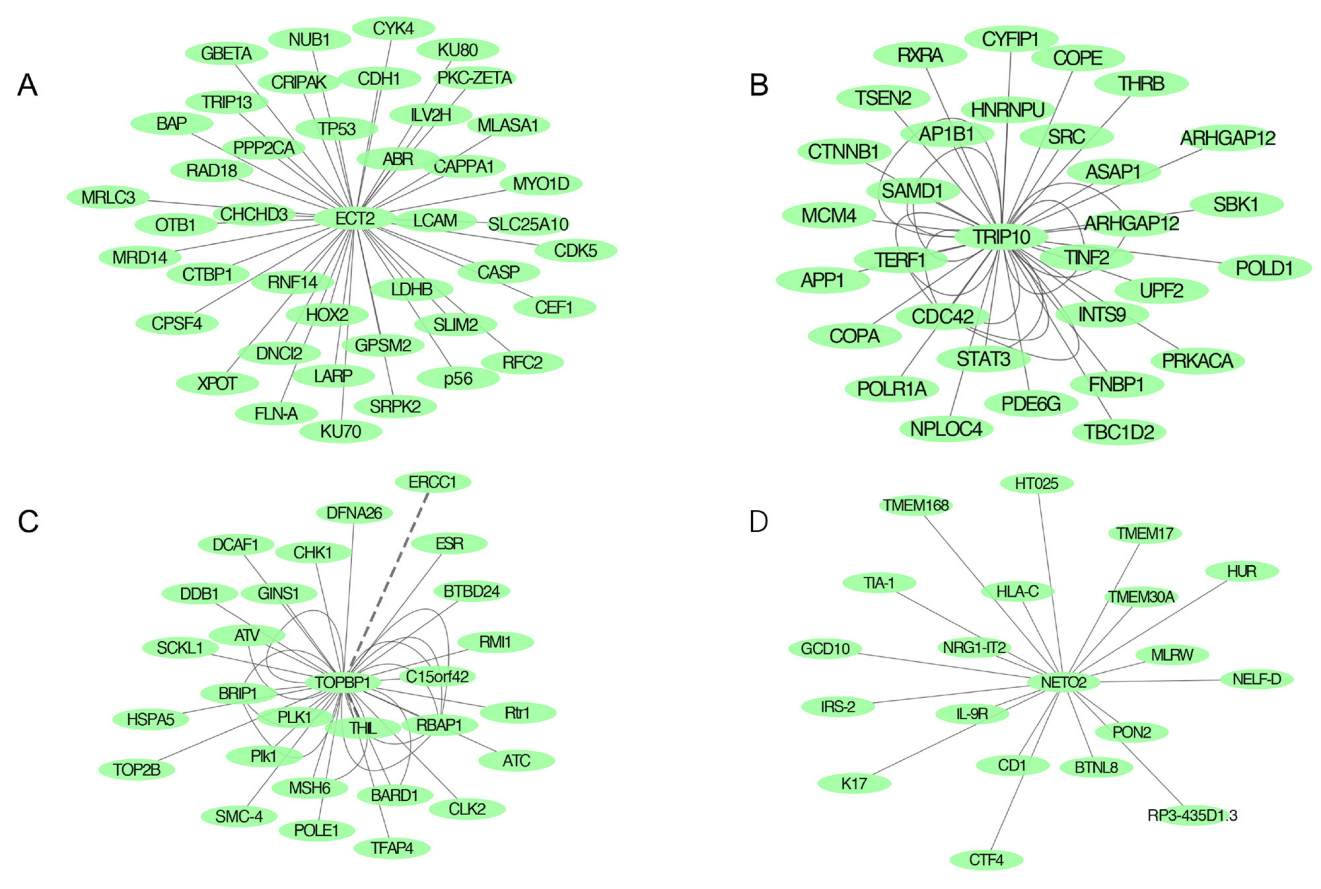
Protein-protein network predicting highly potential interactions with candidate genes based on BioGrid and SRTING databases **(A)** PPI network of ECT2; **(B)** PPI network of TRIP10; **(C)** PPI network of TOPBP1; **(D)** PPI network of NETO2.

Protein–protein interaction (PPI) networks of candidate genes were based on Biogrid and String database (Supplementary Figure 2). A total of 160 proteins might combine with ECT2 protein, which were detected by high-throughput analysis (Figure 7A). Among them, we found several molecules like cyclin-dependent kinase 5 (CDK5), tumor protein P53 (TP53) and E-cadherin (*CDH1*), were important proteins in cancer signal transduction pathway. Additionally, TRIP10 might interact with 57 proteins including Rho GTPase activating protein 17 (ARHGAP17), cell division cycle 42 (CDC42) and signal transducer and activator of transcription 3 (STAT3) in ESCC (Figure 7B). As to TOPBP1, E2F transcription factor1 (E2F1), mediator of DNA-damage checkpoint1 (MDC1) and BRCA1 associated RING domain 1 (BRAD1) was found in the interaction network (Figure 7C).

**Figure 8:**
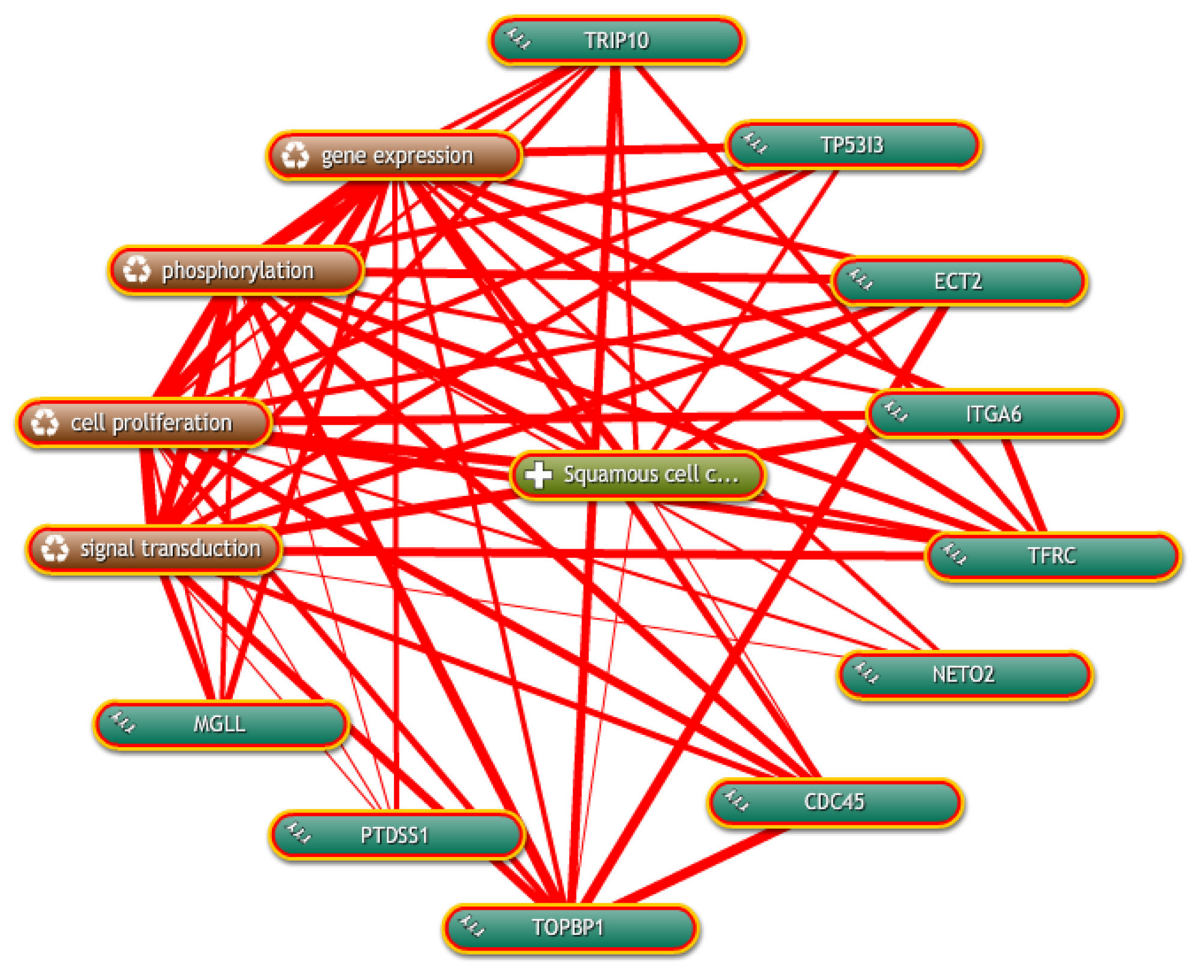
Inter-relationship of candidate genes with ESCC was determined by text mining using Coremine Medical

Coremine Medical mining was performed to identify the potential roles of the 9 candidate genes in ESCC. As shown in Figure 8, there were significant connections between gene expression, phosphorylation, cell proliferation and signal transduction of ESCC with candidate genes. For instance, *TRIP10, TP53I3, ECT2, ITGA6, TFRC, NETO2, TOPBP1* and *PTDSS1* were correlated with cell proliferation in this network.

All genes had at least 4 connections with factors related to ESCC.

### Correlation of CNV and expression of the candidate genes

**Figure 9:**
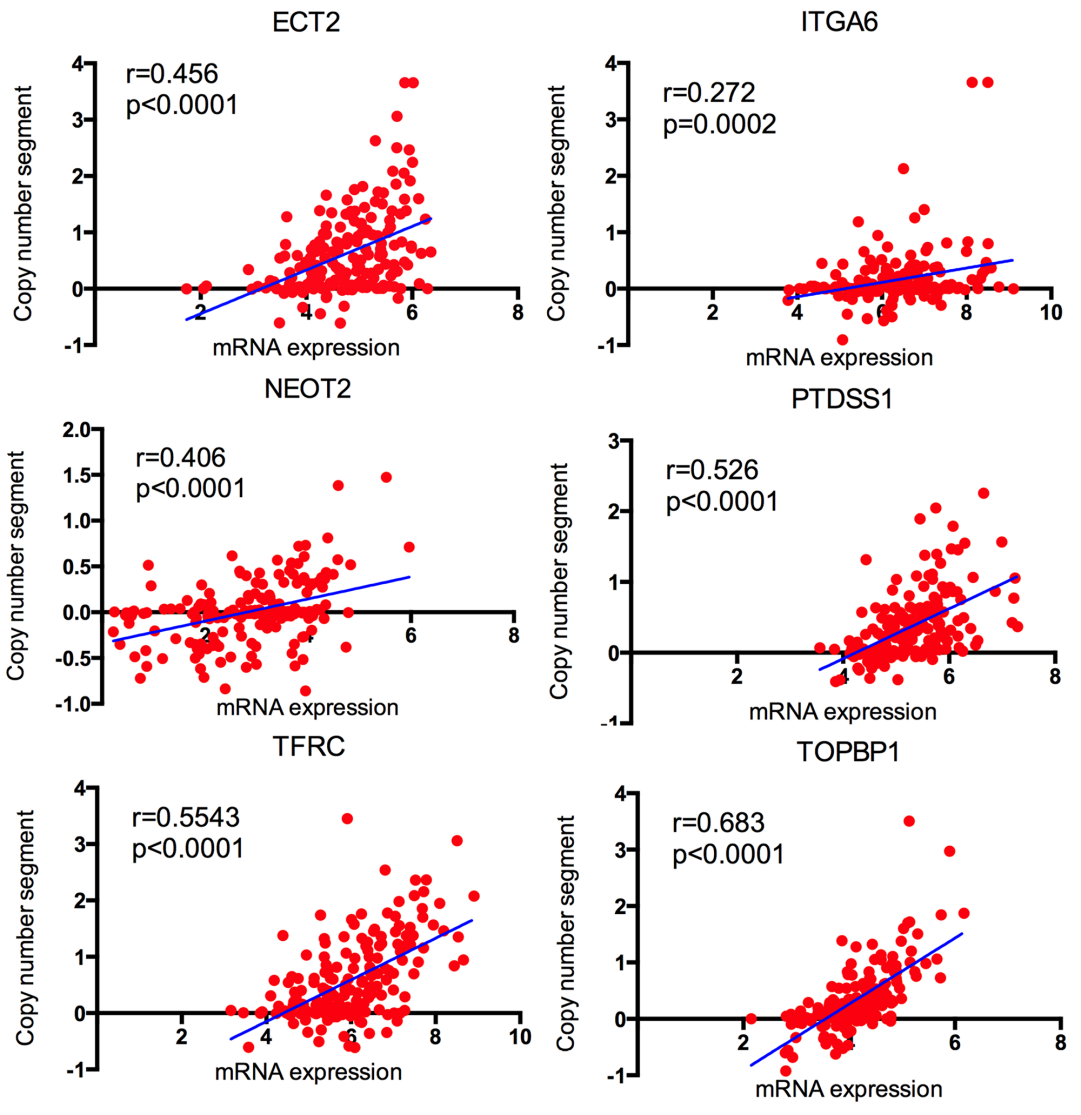
The correlation between copy number segment and the corresponding mRNA expression in TCGA

To investigate the correlations between copy number variation and the expression of candidate genes, TCGA CNV and expression profiles were downloaded. We explored the up/down-regulated genes and amplified/deletion in copy number. By bivariate analysis, 6 up-regulated genes, *ECT2, ITGA6, TFRC, NETO2, TOPBP1* and *PTDSS1* (Figure 9) revealed positive correlation with amplified in copy number. It indicated that up regulated of 6 genes might be resulted from DNA copy number amplification.

## DISCUSSION

ESCC presents high prevalence and mortality in China [3]. Distant metastasis and recurrence are two major lethal issues [2]. The main objective for this study was to discover more powerful and useful biomarkers or therapeutic targets that involved in the pathogenesis of ESCC by utilizing public datasets.

In the present study, we identified 200 deregulated genes between cancer and normal tissues of ESCC by meta-analysis across two independent microarrays with 140 cases. By validating in another ESCC microarray with 179 paired samples, we screened 139 consistently and stably deregulated genes. In addition, survival analysis identified nine candidate genes related to prognosis of ESCC. Furthermore, by conducting a series of bioinformatics analysis, we revealed that nine candidate genes mainly regulated cell cycle and cell-cell adhesion pathways that were closely related with progression of ESCC. Finally, we found that up-regulated of *ECT2* and other five genes were correlated with amplification of copy number. Together, our data clarified nine candidate genes related to progression and prognosis of ESCC and corresponding potential mechanisms.

Among nine candidate genes, *ECT2, TFRC* and *ITGA6* have been explored in ESCC [20]. *ECT2* encodes a guanine nucleotide exchange factor and transforming protein related to Rho-specific exchange factors. Hirata reported to identify *ECT2* as a candidate prognostic biomarker and regulate cancer cell growth [21]. *TFRC* is a cell surface receptor that is necessary for cellular iron uptake by the process of receptor-mediated endocytosis. *TFRC* was identified as a prognostic factor in patients with ESCC and correlated with amplification of the chromosome 3q [22]. *ITGA6* belongs to integrin alpha chain family, which activates with other extracellular matrix proteins. Kwon’s study found that *ITGA6* promoted the proliferation and invasion of ESCC (24042193). These results were consistent with our findings. To further explore the possible molecular mechanisms, we performed GO term analysis and KEGG pathway analysis. We found that the three genes mainly enriched in cell division, cell cycle and cell-cell adhesion pathways. In addition, protein-protein network analyses indicated that ECT2 might interact with other 160 proteins including CDK5, TP53 and E-cadherin. CDK5 is a serine/threonine kinase that controls the cell cycle and proliferation. TP53 encodes a tumor suppressor transcription factor that regulates cell cycle arrest, apoptosis. E-cadherin is a important bio-marker in EMT progression. These results indicated that ECT2 might activate cell cycle, apoptosis and cell invasion by these proteins. Previous studies demonstrated that ECT2 could promote the growth and invasion of esophageal cancer, which were consisted with our results.

Besides above-mentioned genes, we also identified novel candidate genes related to progression of ESCC. TOPBP1 has been reported to accelerate tumor development in multi-cancers [23]. However, little is known to its functions in ESCC. Previous studies reported that TOPBP1 played conserved roles in the initiation of DNA replication and activation of DNA damage checkpoint signaling by serving as scaffolding protein [24]. In our study, TOPBP1 was found to be up regulated in cancer tissues and related to survival. Pathway analysis showed up-regulated TOPBP1 could regulate the mRNA splicing, DNA replication and DNA repair pathways. Protein-protein network showed that TOPBP1 could activate or inhibit with other 50 proteins including E2F1, MDC1 and BARD1. These proteins mainly served in DNA replication and DNA repair pathways, indicating that TOPBP1 might function as an oncogene through these mechanisms. NETO2 is a single-pass transmembrane protein with two extracellular CUB domains. Increasing evidence showed that NETO2 was an oncogene in colorectal carcinoma and hepatocellular carcinoma [25, 26]. One report found that NETO2 was related to rapidly growing hepatocellular carcinoma [26]. Another study pointed out that up-regulation of NETO2 expression correlated with tumor progression and poor prognosis in colorectal carcinoma [25]. NETO2 was a prognostic biomarker and participated in cell division and cell proliferation pathways by our analysis. This notion is in line with published studies. Among the down-regulated genes, TRIP10, MGLL and TP53I3 were well investigated in previous studies. TRIP10, also named CIP4, was related to lung cancer and chronic lymphocytic leukemia [27, 28]. It was defined as functional interactions during biogenesis of epithelial junctions. By our analysis, TRIP10 was correlated with cell-cell adhesion, regulation of Rho protein signal transduction and positive regulation of GTPase activity pathways, suggesting potential roles in cell invasion and migration of ESCC. TP53I3 was correlated with melanoma and glioblastoma [29, 30]. Intriguingly, TP53I3 was up regulated in these cancers. It functioned as oncogenes in glioblastoma and papillary thyroid cancer [31]. However, TP53I3 was down regulated in our analysis, indicating a unique role in ESCC. MGLL is a serine hydrolase of the AB hydrolase superfamily. Published studies demonstrated that MGLL was correlated with colorectal cancer and gastric cancer [32, 33]. In current analyses, we identified that MGLL was correlated with survival and participated multi-pathways in ESCC.

The present study facilitated access and interpretation of candidate genes related to progression and prognosis of ESCC. However, some limitations should be mentioned. Our data were analyzed by bioinformatics. Experiment data are needed to support our analyses. In addition, only two studies were included in the analysis. The sample size was too small. Studies with large samples were needed to further validate. Furthermore, several studies built sub-network for cancer prognosis by systematically analyzing multi-dimensional cancer genomics data [34, 35]. However, there was lack of epigenetic and DNA mutations in our analyses. Integrated analyses of genetic, epigenetic and proteomic could be included in further study.

In summary, the current study demonstrated nine genes related to progression and prognosis of ESCC using the public portals and bioinformatics, which provided novel prognostic biomarkers and contributed to a better understanding of cancer molecular mechanisms. This will ultimately accelerate the translation of bench work into bedside.

## MATERIALS AND METHODS

### Meta-analysis & data source

We searched eligible ESCC microarray datasets from Oncomine platform by adding filters like mRNA data, clinical specimens and cancer vs normal. To identify candidate genes, meta-analysis of gene expression profiles was performed across ESCC datasets in ONCOMINE 4.5 (https://www.oncomine.org/resource/). The deregulated genes were ranked by median ranks across the selected analyses and displayed corresponding p-values. GSE53625 and corresponding survival information were downloaded from GEO profiles databases for validation (http://www.ncbi.nlm.nih.gov/geoprofiles/)[36]. The limma R package was also utilized to identify the deregulated gene expression between cancer and normal. The level 3 TCGA data TCGA_ESCA_GSNP6noCNV-2015-02-24 (delete germline CNV) and TCGA_ESCA_exp_HiSeq-2015-02-24 were downloaded from Cancer Browser (http://www.genome-cancer.ucsc.edu).

### Survival analysis

Kaplan-Meier method was performed to identify genes correlated with survival. Log rank test was used to evaluate the significant differences. The cutoff of expression value was defined as median expression level in each gene expression profile. Survival curves were constructed using GraphPad Prism v6.0 (GraphPad Software, San Diego, CA, USA). Hazard ratio and 95% confidence interval were also calculated.

### Bioinformatics analysis

Co-expression analysis was performed based on the functions of Oncomine. By selected a co-expression analysis in the datasets tab, the system displayed a co-expression heat map. Typed the target gene into search bar, co-expression genes were displayed by their correlations to target gene. A correlation value closer to 1.0 indicates genes that are more highly correlated with the selected gene of interest. The list of co-expression genes were submitted to a functional annotation tool named DAVID Bioinformatics Resources 6.8 for KEGG pathway and GO biological process enrichment analysis (https://david.ncifcrf.gov). Protein-protein interaction was conducted in Cytoscape public databases and GeneMANIA plug-in (http://www.genemania.org/). Text mining was used to display potential roles in ESCC by the Coremine Medical online database (http://www.coremine.com/ medical/). Gene Set Enrichment Analysis (GSEA) was performed to determine whether a priori defined set of genes shows statistically significant, consistent differences between two biological states [37].

### Expression and CNV analysis

There were 184 esophageal cancer samples in DNA copy number variant (CNV) of TCGA and 198 esophageal cancer samples in expression profile of TCGA. Among them, a total of 183 samples were matched between expression and CNV. The correlation between the CNVs and expression of genes were evaluated by bivariate correlations. Pearson correlation was used. Significant differences were defined as p<0.05.

## SUPPLEMENTARY MATERIALS FIGURES AND TABLES









## Abbreviations

ESCC: esophageal squamous cell carcinoma
TCGA: The Cancer Genome Atlas
GEO: Gene Expression Omnibus
CNV: copy number variant
GO: Gene Ontology
KEGG: Kyoto Encyclopedia of Genes and Genomes
NCCR: National Central Cancer Registry of China
