# Supplementary material for: Microarray analyses reveal genes related to progression and prognosis of esophageal squamous cell carcinoma
Source: Oncotarget. 2017 Aug 12;8(45):78838–50. doi: 10.18632/oncotarget.20232 (PMC5668002; doi:10.18632/oncotarget.20232)
Supplement: Supplementary file 1 [file oncotarget-08-78838-s001.pdf]

Microarray analyses reveal genes related to progression and prognosis of esophageal squamous cell carcinoma

SUPPLEMENTARY MATERIALS

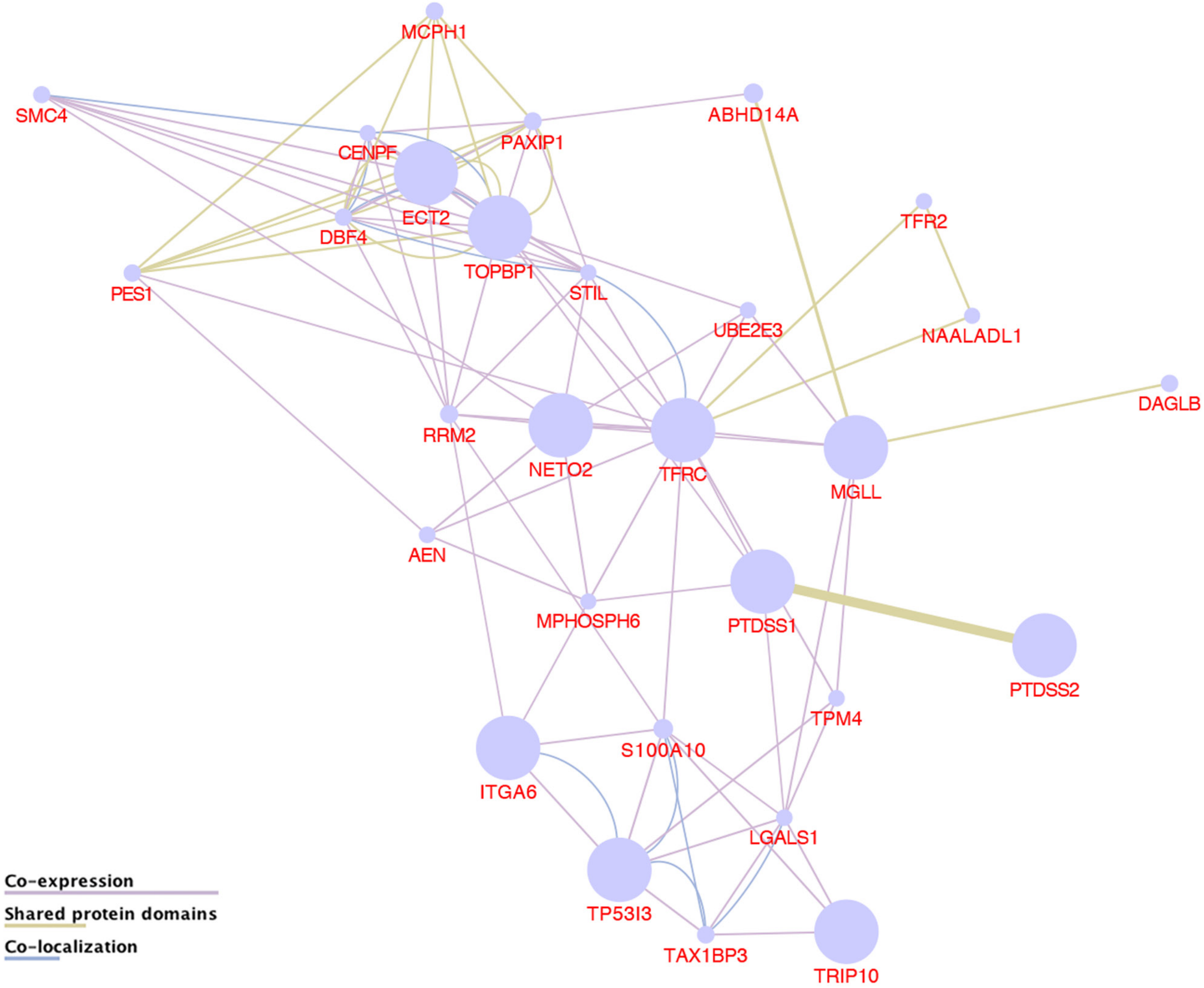

Supplementary Figure 1: Inter-relationship between candidate genes and other key molecules by GeneMiana tool.

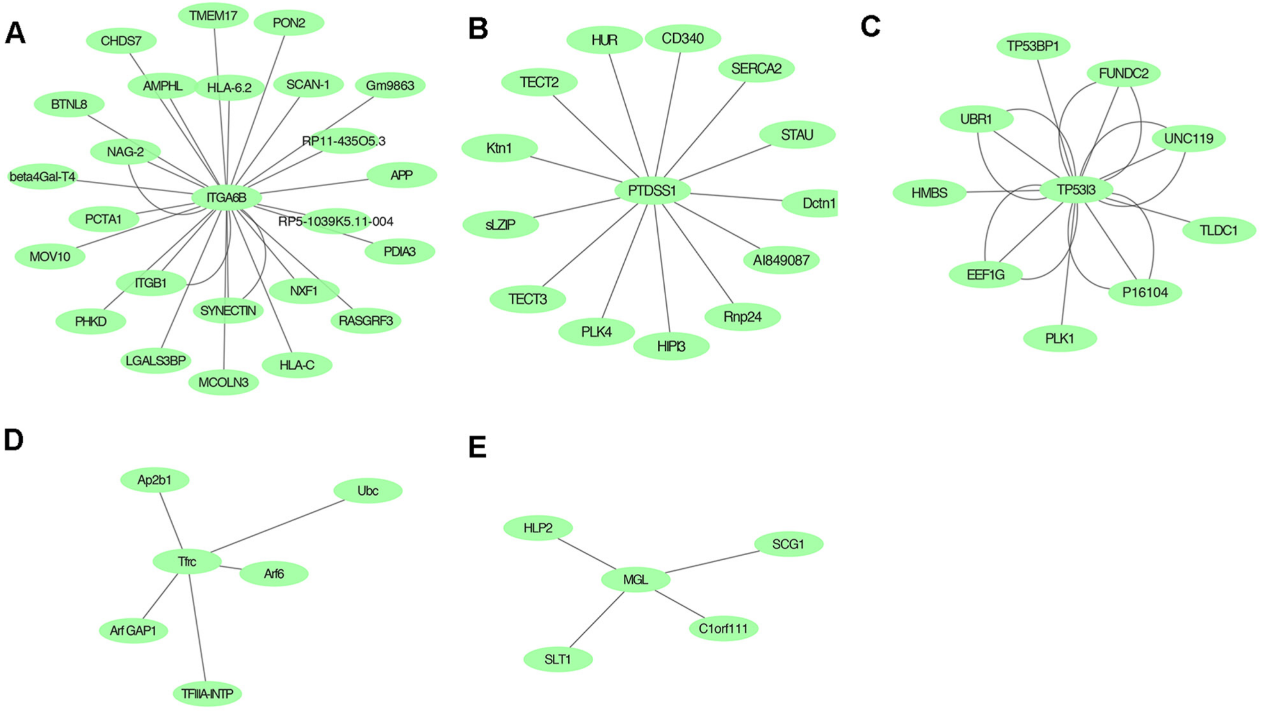

AQ:2 **Supplementary Figure 2: Protein-protein network predicting highly potential interactions with other five candidate genes based on BioGrid and SRTING databases.**

Supplementary Table 1: Up-regulated genes by meta-analysis

See Supplementary File 1

Supplementary Table2: Down-regulated genes by meta-analysis

See Supplementary File 2

Supplementary Table 3: Relationships between of candidate genes

See Supplementary File 3
