# Supplementary material for: Microarray analyses reveal genes related to progression and prognosis of esophageal squamous cell carcinoma
Source: Oncotarget. 2017 Aug 12;8(45):78838–50. doi: 10.18632/oncotarget.20232 (PMC5668002; doi:10.18632/oncotarget.20232)
Supplement: Supplementary file 2 [file oncotarget-08-78838-s002.docx]

**Supplementary Table 1: Up-regulated genes by meta-analysis**

| **Gene symbol** | Hu’s ESCC cohort | | Su’s ESCC cohort | | Median | |
| --- | --- | --- | --- | --- | --- | --- |
|  | **Gene rank** | **P-value** | **Gene rank** | **P-value** | **Gene rank** | **P-value** |
| ECT2 | 3 | 1.74E-12 | 3 | 1.20E-30 | 3 | 8.68E-13 |
| MCM2 | 13 | 2.39E-11 | 7 | 7.80E-28 | 10 | 1.19E-11 |
| CDK1 | 16 | 3.52E-11 | 10 | 1.54E-26 | 13 | 1.76E-11 |
| LAPTM4B | 14 | 2.61E-11 | 25 | 5.28E-24 | 19.5 | 1.31E-11 |
| TCF3 | 4 | 1.82E-12 | 44 | 2.57E-22 | 24 | 9.08E-13 |
| CBX3 | 43 | 3.21E-10 | 6 | 3.75E-29 | 24.5 | 1.60E-10 |
| KIF14 | 37 | 2.43E-10 | 13 | 1.34E-25 | 25 | 1.22E-10 |
| KIF4A | 41 | 2.84E-10 | 11 | 2.99E-26 | 26 | 1.42E-10 |
| GINS1 | 23 | 8.86E-11 | 40 | 1.55E-22 | 31.5 | 4.43E-11 |
| PLAU | 53 | 5.67E-10 | 12 | 1.27E-25 | 32.5 | 2.83E-10 |
| NEK2 | 1 | 1.53E-12 | 67 | 3.57E-21 | 34 | 7.64E-13 |
| UBE2C | 52 | 5.26E-10 | 20 | 1.14E-24 | 36 | 2.63E-10 |
| ACTL6A | 58 | 6.70E-10 | 16 | 7.24E-25 | 37 | 3.35E-10 |
| FZD6 | 36 | 2.38E-10 | 42 | 2.18E-22 | 39 | 1.19E-10 |
| HOXB7 | 17 | 4.08E-11 | 63 | 1.93E-21 | 40 | 2.04E-11 |
| RFC4 | 82 | 1.89E-09 | 5 | 1.15E-29 | 43.5 | 9.45E-10 |
| CEP55 | 81 | 1.86E-09 | 14 | 1.49E-25 | 47.5 | 9.31E-10 |
| SR140 | 55 | 6.30E-10 | 46 | 3.24E-22 | 50.5 | 3.15E-10 |
| ARL6IP1 | 25 | 1.03E-10 | 82 | 1.36E-20 | 53.5 | 5.13E-11 |
| TIMELESS | 39 | 2.50E-10 | 68 | 4.52E-21 | 53.5 | 1.25E-10 |
| MARCKSL1 | 88 | 2.55E-09 | 22 | 1.57E-24 | 55 | 1.28E-09 |
| TOP2A | 77 | 1.59E-09 | 36 | 6.04E-23 | 56.5 | 7.95E-10 |
| TFRC | 62 | 9.26E-10 | 53 | 5.18E-22 | 57.5 | 4.63E-10 |
| SNAI2 | 100 | 3.61E-09 | 18 | 8.68E-25 | 59 | 1.80E-09 |
| TOPBP1 | 28 | 1.24E-10 | 96 | 4.76E-20 | 62 | 6.18E-11 |
| TRIP13 | 46 | 3.56E-10 | 85 | 2.25E-20 | 65.5 | 1.78E-10 |
| BUB1B | 113 | 5.98E-09 | 24 | 3.07E-24 | 68.5 | 2.99E-09 |
| HSP90B1 | 83 | 2.17E-09 | 55 | 7.62E-22 | 69 | 1.09E-09 |
| DLGAP5 | 49 | 4.55E-10 | 90 | 3.04E-20 | 69.5 | 2.28E-10 |
| SPP1 | 101 | 3.89E-09 | 41 | 1.99E-22 | 71 | 1.94E-09 |
| MEST | 86 | 2.36E-09 | 59 | 1.11E-21 | 72.5 | 1.18E-09 |
| PLXNA1 | 136 | 1.49E-08 | 9 | 1.58E-27 | 72.5 | 7.45E-09 |
| RNPS1 | 34 | 1.89E-10 | 112 | 8.38E-20 | 73 | 9.44E-11 |
| FNDC3B | 6 | 4.61E-12 | 141 | 3.37E-19 | 73.5 | 2.31E-12 |
| MBD4 | 45 | 3.49E-10 | 107 | 6.82E-20 | 76 | 1.74E-10 |
| MTHFD2 | 5 | 2.06E-12 | 148 | 5.51E-19 | 76.5 | 1.03E-12 |
| ATP2C1 | 132 | 1.20E-08 | 30 | 2.74E-23 | 81 | 6.00E-09 |
| TTK | 22 | 8.52E-11 | 142 | 3.82E-19 | 82 | 4.26E-11 |
| FSCN1 | 107 | 4.90E-09 | 60 | 1.16E-21 | 83.5 | 2.45E-09 |
| CDKN3 | 69 | 1.06E-09 | 102 | 5.71E-20 | 85.5 | 5.31E-10 |
| HJURP | 40 | 2.67E-10 | 132 | 2.09E-19 | 86 | 1.33E-10 |
| MCM4 | 50 | 4.58E-10 | 122 | 1.56E-19 | 86 | 2.29E-10 |
| AURKA | 35 | 2.14E-10 | 138 | 2.97E-19 | 86.5 | 1.07E-10 |
| KIF2C | 117 | 8.31E-09 | 56 | 7.79E-22 | 86.5 | 4.16E-09 |
| POLR2H | 12 | 2.28E-11 | 162 | 9.91E-19 | 87 | 1.14E-11 |
| BIRC5 | 134 | 1.35E-08 | 43 | 2.48E-22 | 88.5 | 6.76E-09 |
| YEATS2 | 48 | 4.17E-10 | 137 | 2.91E-19 | 92.5 | 2.09E-10 |
| MFAP2 | 175 | 3.44E-08 | 17 | 7.35E-25 | 96 | 1.72E-08 |
| PTK2 | 87 | 2.49E-09 | 110 | 8.01E-20 | 98.5 | 1.25E-09 |
| PCNA | 103 | 4.39E-09 | 95 | 3.82E-20 | 99 | 2.19E-09 |
| HMGB3 | 130 | 1.18E-08 | 70 | 5.17E-21 | 100 | 5.89E-09 |
| GTF2E1 | 91 | 2.74E-09 | 111 | 8.14E-20 | 101 | 1.37E-09 |
| KIF20A | 139 | 1.57E-08 | 65 | 2.07E-21 | 102 | 7.83E-09 |
| TPX2 | 178 | 3.66E-08 | 29 | 2.19E-23 | 103.5 | 1.83E-08 |
| FUS | 75 | 1.42E-09 | 133 | 2.13E-19 | 104 | 7.09E-10 |
| RPN1 | 161 | 2.47E-08 | 48 | 4.41E-22 | 104.5 | 1.23E-08 |
| BUB1 | 126 | 1.04E-08 | 86 | 2.36E-20 | 106 | 5.19E-09 |
| LEPRE1 | 38 | 2.48E-10 | 178 | 2.48E-18 | 108 | 1.24E-10 |
| HOMER3 | 145 | 1.83E-08 | 72 | 5.55E-21 | 108.5 | 9.17E-09 |
| DUSP12 | 8 | 8.22E-12 | 210 | 6.44E-18 | 109 | 4.11E-12 |
| DNMT1 | 164 | 2.83E-08 | 58 | 1.02E-21 | 111 | 1.41E-08 |
| RUVBL1 | 197 | 5.13E-08 | 27 | 1.62E-23 | 112 | 2.56E-08 |
| VCAN | 140 | 1.61E-08 | 88 | 2.50E-20 | 114 | 8.07E-09 |
| FEN1 | 149 | 2.04E-08 | 79 | 8.74E-21 | 114 | 1.02E-08 |
| ATAD2 | 65 | 1.01E-09 | 166 | 1.29E-18 | 115.5 | 5.03E-10 |
| SULF1 | 155 | 2.26E-08 | 77 | 7.73E-21 | 116 | 1.13E-08 |
| ADAR | 84 | 2.21E-09 | 150 | 6.00E-19 | 117 | 1.10E-09 |
| MMP12 | 9 | 9.00E-12 | 226 | 1.18E-17 | 117.5 | 4.50E-12 |
| KIF18B | 74 | 1.39E-09 | 161 | 9.36E-19 | 117.5 | 6.93E-10 |
| HNRNPA2B1 | 26 | 1.17E-10 | 215 | 7.73E-18 | 120.5 | 5.85E-11 |
| FOXM1 | 223 | 8.26E-08 | 26 | 1.46E-23 | 124.5 | 4.13E-08 |
| AGRN | 233 | 8.68E-08 | 23 | 2.61E-24 | 128 | 4.34E-08 |
| IGF2BP2 | 20 | 6.07E-11 | 237 | 1.73E-17 | 128.5 | 3.04E-11 |
| STIL | 125 | 9.42E-09 | 136 | 2.80E-19 | 130.5 | 4.71E-09 |
| MET | 190 | 4.64E-08 | 76 | 6.88E-21 | 133 | 2.32E-08 |
| COL7A1 | 206 | 6.00E-08 | 62 | 1.60E-21 | 134 | 3.00E-08 |
| MAD2L1 | 129 | 1.15E-08 | 140 | 3.28E-19 | 134.5 | 5.77E-09 |
| POLR2K | 187 | 4.33E-08 | 83 | 1.63E-20 | 135 | 2.16E-08 |
| ITPR3 | 237 | 9.00E-08 | 37 | 6.24E-23 | 137 | 4.50E-08 |
| POSTN | 227 | 8.55E-08 | 50 | 4.85E-22 | 138.5 | 4.27E-08 |
| SNRPG | 184 | 4.18E-08 | 97 | 4.96E-20 | 140.5 | 2.09E-08 |
| FANCI | 173 | 3.39E-08 | 116 | 1.03E-19 | 144.5 | 1.70E-08 |
| SLC25A32 | 211 | 6.16E-08 | 78 | 8.04E-21 | 144.5 | 3.08E-08 |
| THY1 | 94 | 3.00E-09 | 203 | 4.96E-18 | 148.5 | 1.50E-09 |
| PRKDC | 218 | 6.82E-08 | 80 | 1.11E-20 | 149 | 3.41E-08 |
| CKS2 | 243 | 9.78E-08 | 57 | 8.77E-22 | 150 | 4.89E-08 |
| H2AFZ | 30 | 1.31E-10 | 272 | 4.96E-17 | 151 | 6.55E-11 |
| NETO2 | 180 | 3.80E-08 | 125 | 1.60E-19 | 152.5 | 1.90E-08 |
| PRC1 | 264 | 1.31E-07 | 45 | 2.85E-22 | 154.5 | 6.55E-08 |
| MMP1 | 307 | 2.58E-07 | 8 | 7.99E-28 | 157.5 | 1.29E-07 |
| RAI14 | 142 | 1.77E-08 | 174 | 2.21E-18 | 158 | 8.86E-09 |
| ADCY3 | 188 | 4.50E-08 | 128 | 1.79E-19 | 158 | 2.25E-08 |
| STMN1 | 203 | 5.76E-08 | 113 | 8.39E-20 | 158 | 2.88E-08 |
| ATP1B3 | 21 | 6.24E-11 | 297 | 8.54E-17 | 159 | 3.12E-11 |
| NUSAP1 | 128 | 1.13E-08 | 191 | 3.56E-18 | 159.5 | 5.63E-09 |
| ATP6V1C1 | 221 | 8.19E-08 | 101 | 5.69E-20 | 161 | 4.09E-08 |
| PBK | 61 | 9.07E-10 | 264 | 4.10E-17 | 162.5 | 4.54E-10 |
| CSE1L | 89 | 2.66E-09 | 238 | 1.79E-17 | 163.5 | 1.33E-09 |
| MORC2 | 11 | 1.88E-11 | 320 | 1.99E-16 | 165.5 | 9.41E-12 |
| SLC20A1 | 263 | 1.31E-07 | 73 | 5.83E-21 | 168 | 6.54E-08 |
| CPSF6 | 213 | 6.28E-08 | 127 | 1.69E-19 | 170 | 3.14E-08 |
| ATR | 202 | 5.57E-08 | 139 | 3.09E-19 | 170.5 | 2.78E-08 |
| DTL | 277 | 1.52E-07 | 64 | 2.01E-21 | 170.5 | 7.61E-08 |
| ITGA6 | 194 | 4.98E-08 | 151 | 6.03E-19 | 172.5 | 2.49E-08 |
| PARL | 33 | 1.74E-10 | 313 | 1.67E-16 | 173 | 8.68E-11 |
| WDHD1 | 10 | 1.69E-11 | 338 | 3.26E-16 | 174 | 8.46E-12 |
| TROAP | 98 | 3.36E-09 | 250 | 2.80E-17 | 174 | 1.68E-09 |
| DPY19L4 | 177 | 3.55E-08 | 180 | 2.65E-18 | 178.5 | 1.77E-08 |
| SLC39A6 | 76 | 1.45E-09 | 284 | 6.21E-17 | 180 | 7.27E-10 |
| HSP90AA1 | 144 | 1.79E-08 | 220 | 1.01E-17 | 182 | 8.93E-09 |
| LAMB3 | 208 | 6.12E-08 | 156 | 7.90E-19 | 182 | 3.06E-08 |
| MYBL2 | 54 | 5.94E-10 | 311 | 1.39E-16 | 182.5 | 2.97E-10 |
| DDX39 | 122 | 9.12E-09 | 243 | 2.19E-17 | 182.5 | 4.56E-09 |
| NUP107 | 168 | 2.92E-08 | 201 | 4.24E-18 | 184.5 | 1.46E-08 |
| NCAPG | 111 | 5.53E-09 | 260 | 3.91E-17 | 185.5 | 2.76E-09 |
| DDX11 | 205 | 5.95E-08 | 168 | 1.51E-18 | 186.5 | 2.98E-08 |
| LRRC8D | 255 | 1.16E-07 | 118 | 1.16E-19 | 186.5 | 5.78E-08 |
| HLTF | 24 | 9.80E-11 | 350 | 5.04E-16 | 187 | 4.90E-11 |
| MCM6 | 353 | 4.32E-07 | 21 | 1.15E-24 | 187 | 2.16E-07 |
| PSME4 | 300 | 2.36E-07 | 75 | 6.16E-21 | 187.5 | 1.18E-07 |
| CENPM | 63 | 9.37E-10 | 316 | 1.79E-16 | 189.5 | 4.68E-10 |
| PDIA5 | 141 | 1.70E-08 | 239 | 2.00E-17 | 190 | 8.51E-09 |
| SSRP1 | 204 | 5.83E-08 | 177 | 2.44E-18 | 190.5 | 2.92E-08 |
| SOX4 | 262 | 1.26E-07 | 120 | 1.46E-19 | 191 | 6.29E-08 |
| NCAPG2 | 279 | 1.60E-07 | 103 | 6.09E-20 | 191 | 8.01E-08 |
| NUP155 | 288 | 2.02E-07 | 98 | 5.00E-20 | 193 | 1.01E-07 |
| C1orf107 | 105 | 4.58E-09 | 282 | 6.03E-17 | 193.5 | 2.29E-09 |
| MCM5 | 298 | 2.32E-07 | 93 | 3.60E-20 | 195.5 | 1.16E-07 |
| ERH | 337 | 3.77E-07 | 54 | 5.60E-22 | 195.5 | 1.89E-07 |
| SLC33A1 | 239 | 9.05E-08 | 153 | 6.32E-19 | 196 | 4.52E-08 |
| HNRNPU | 68 | 1.05E-09 | 329 | 2.37E-16 | 198.5 | 5.26E-10 |
| PTDSS1 | 316 | 3.00E-07 | 81 | 1.12E-20 | 198.5 | 1.50E-07 |
| KPNA2 | 85 | 2.32E-09 | 314 | 1.73E-16 | 199.5 | 1.16E-09 |
| ORC6L | 2 | 1.71E-12 | 403 | 1.96E-15 | 202.5 | 8.56E-13 |
| DHX9 | 70 | 1.14E-09 | 335 | 2.91E-16 | 202.5 | 5.72E-10 |
| CCT5 | 301 | 2.36E-07 | 105 | 6.72E-20 | 203 | 1.18E-07 |
| BID | 247 | 1.07E-07 | 160 | 9.29E-19 | 203.5 | 5.35E-08 |
| CCNB2 | 32 | 1.72E-10 | 376 | 1.06E-15 | 204 | 8.60E-11 |
| CDH11 | 335 | 3.76E-07 | 74 | 5.94E-21 | 204.5 | 1.88E-07 |
| AURKB | 230 | 8.61E-08 | 181 | 2.69E-18 | 205.5 | 4.31E-08 |
| EPCAM | 7 | 8.12E-12 | 405 | 2.06E-15 | 206 | 4.06E-12 |
| PDIA4 | 250 | 1.09E-07 | 163 | 1.00E-18 | 206.5 | 5.46E-08 |
| PSMB4 | 123 | 9.36E-09 | 292 | 7.28E-17 | 207.5 | 4.68E-09 |
| RAD51 | 151 | 2.11E-08 | 268 | 4.33E-17 | 209.5 | 1.05E-08 |
| PARP1 | 210 | 6.14E-08 | 209 | 6.35E-18 | 209.5 | 3.07E-08 |
| TMEM194A | 80 | 1.79E-09 | 340 | 3.50E-16 | 210 | 8.96E-10 |
| HEATR1 | 341 | 3.87E-07 | 84 | 1.84E-20 | 212.5 | 1.93E-07 |
| GMPS | 313 | 2.83E-07 | 114 | 9.70E-20 | 213.5 | 1.42E-07 |
| HSPD1 | 378 | 6.12E-07 | 49 | 4.55E-22 | 213.5 | 3.06E-07 |
| HSPBAP1 | 147 | 2.00E-08 | 285 | 6.57E-17 | 216 | 1.00E-08 |
| TRAM2 | 60 | 9.03E-10 | 374 | 1.03E-15 | 217 | 4.51E-10 |
| MTERFD1 | 232 | 8.67E-08 | 202 | 4.92E-18 | 217 | 4.34E-08 |
| FTSJ2 | 349 | 4.12E-07 | 87 | 2.48E-20 | 218 | 2.06E-07 |
| ENY2 | 172 | 3.33E-08 | 265 | 4.23E-17 | 218.5 | 1.66E-08 |
| PFDN2 | 249 | 1.08E-07 | 193 | 3.73E-18 | 221 | 5.42E-08 |
| SLC16A1 | 254 | 1.13E-07 | 190 | 3.51E-18 | 222 | 5.64E-08 |
| CDC20 | 315 | 2.92E-07 | 130 | 1.98E-19 | 222.5 | 1.46E-07 |
| ILF3 | 270 | 1.47E-07 | 179 | 2.48E-18 | 224.5 | 7.35E-08 |
| DDX12 | 345 | 4.08E-07 | 104 | 6.44E-20 | 224.5 | 2.04E-07 |
| SPARC | 299 | 2.35E-07 | 152 | 6.12E-19 | 225.5 | 1.18E-07 |
| CDC25B | 96 | 3.09E-09 | 367 | 8.38E-16 | 231.5 | 1.55E-09 |
| MCM7 | 167 | 2.87E-08 | 298 | 8.61E-17 | 232.5 | 1.43E-08 |
| CCDC21 | 176 | 3.52E-08 | 295 | 8.44E-17 | 235.5 | 1.76E-08 |
| H2AFV | 282 | 1.76E-07 | 197 | 4.01E-18 | 239.5 | 8.79E-08 |
| STK3 | 181 | 3.95E-08 | 303 | 1.00E-16 | 242 | 1.98E-08 |
| MRPL3 | 393 | 7.13E-07 | 92 | 3.34E-20 | 242.5 | 3.57E-07 |
| ANP32E | 66 | 1.02E-09 | 420 | 2.92E-15 | 243 | 5.09E-10 |
| PPPDE1 | 317 | 3.00E-07 | 169 | 1.52E-18 | 243 | 1.50E-07 |
| GMNN | 454 | 1.41E-06 | 32 | 3.71E-23 | 243 | 7.04E-07 |
| CENPA | 235 | 8.93E-08 | 254 | 3.36E-17 | 244.5 | 4.47E-08 |
| VOPP1 | 391 | 7.07E-07 | 100 | 5.68E-20 | 245.5 | 3.54E-07 |
| SNX10 | 296 | 2.25E-07 | 199 | 4.04E-18 | 247.5 | 1.13E-07 |
| KIAA0406 | 189 | 4.58E-08 | 310 | 1.24E-16 | 249.5 | 2.29E-08 |
| SLC39A14 | 112 | 5.75E-09 | 392 | 1.46E-15 | 252 | 2.87E-09 |
| ENAH | 115 | 7.83E-09 | 396 | 1.61E-15 | 255.5 | 3.92E-09 |
| RACGAP1 | 260 | 1.22E-07 | 251 | 3.07E-17 | 255.5 | 6.11E-08 |
| ACLY | 466 | 1.49E-06 | 47 | 3.63E-22 | 256.5 | 7.44E-07 |
| CDC45 | 214 | 6.31E-08 | 300 | 8.79E-17 | 257 | 3.15E-08 |
| COL11A1 | 207 | 6.02E-08 | 308 | 1.14E-16 | 257.5 | 3.01E-08 |
| MSH6 | 392 | 7.09E-07 | 131 | 2.07E-19 | 261.5 | 3.55E-07 |
| GINS2 | 165 | 2.83E-08 | 361 | 7.22E-16 | 263 | 1.42E-08 |
| MYO10 | 193 | 4.80E-08 | 334 | 2.90E-16 | 263.5 | 2.40E-08 |
| MINPP1 | 200 | 5.44E-08 | 327 | 2.27E-16 | 263.5 | 2.72E-08 |
| SUPT16H | 360 | 4.71E-07 | 171 | 1.55E-18 | 265.5 | 2.35E-07 |
| DCTPP1 | 410 | 8.99E-07 | 124 | 1.58E-19 | 267 | 4.50E-07 |
| TMEM184B | 323 | 3.16E-07 | 213 | 7.24E-18 | 268 | 1.58E-07 |
| ZNF281 | 402 | 8.14E-07 | 134 | 2.32E-19 | 268 | 4.07E-07 |
| SLC38A6 | 356 | 4.48E-07 | 183 | 2.76E-18 | 269.5 | 2.24E-07 |
| COL1A1 | 537 | 2.46E-06 | 2 | 1.18E-30 | 269.5 | 1.23E-06 |
| LPCAT1 | 71 | 1.16E-09 | 476 | 6.89E-15 | 273.5 | 5.80E-10 |
| CDK2 | 311 | 2.74E-07 | 236 | 1.55E-17 | 273.5 | 1.37E-07 |
| MMP11 | 196 | 5.07E-08 | 352 | 5.09E-16 | 274 | 2.53E-08 |
| UMPS | 154 | 2.25E-08 | 395 | 1.48E-15 | 274.5 | 1.12E-08 |
| RPN2 | 435 | 1.17E-06 | 115 | 9.95E-20 | 275 | 5.83E-07 |
| VRK1 | 325 | 3.33E-07 | 227 | 1.19E-17 | 276 | 1.67E-07 |
| DNAJC2 | 274 | 1.50E-07 | 279 | 5.76E-17 | 276.5 | 7.48E-08 |
| TGS1 | 73 | 1.31E-09 | 487 | 8.87E-15 | 280 | 6.57E-10 |
| NASP | 219 | 7.25E-08 | 346 | 3.99E-16 | 282.5 | 3.63E-08 |
| GTSE1 | 146 | 1.92E-08 | 421 | 2.94E-15 | 283.5 | 9.62E-09 |
| CCNB1 | 460 | 1.45E-06 | 108 | 6.87E-20 | 284 | 7.23E-07 |
