# Supplementary material for: Microarray analyses reveal genes related to progression and prognosis of esophageal squamous cell carcinoma
Source: Oncotarget. 2017 Aug 12;8(45):78838–50. doi: 10.18632/oncotarget.20232 (PMC5668002; doi:10.18632/oncotarget.20232)
Supplement: Supplementary file 3 [file oncotarget-08-78838-s003.docx]

**Supplementary Table2: Down-regulated genes by meta-analysis**

| **Gene symbol** | Hu’s ESCC cohort | | Su’s ESCC cohort | | Median | |
| --- | --- | --- | --- | --- | --- | --- |
|  | **Gene rank** | **P-value** | **Gene rank** | **P-value** | **Gene rank** | **P-value** |
| UBL3 | 3 | 1.63E-17 | 2 | 1.81E-25 | 2.5 | 8.14E-18 |
| PPP1R3C | 9 | 6.22E-16 | 20 | 8.94E-23 | 14.5 | 3.11E-16 |
| KAT2B | 31 | 5.14E-14 | 7 | 9.95E-25 | 19 | 2.57E-14 |
| ENDOU | 2 | 1.03E-17 | 49 | 3.49E-20 | 25.5 | 5.17E-18 |
| RMND5B | 30 | 5.01E-14 | 21 | 8.94E-23 | 25.5 | 2.51E-14 |
| SORT1 | 26 | 2.45E-14 | 27 | 4.12E-22 | 26.5 | 1.23E-14 |
| CRYL1 | 50 | 6.13E-13 | 4 | 6.32E-25 | 27 | 3.06E-13 |
| IL1RN | 7 | 5.03E-16 | 50 | 3.86E-20 | 28.5 | 2.52E-16 |
| CRISP3 | 20 | 8.05E-15 | 44 | 1.75E-20 | 32 | 4.03E-15 |
| ETFDH | 38 | 1.05E-13 | 28 | 6.97E-22 | 33 | 5.26E-14 |
| NUCB2 | 47 | 4.27E-13 | 19 | 6.20E-23 | 33 | 2.13E-13 |
| ABR | 22 | 1.25E-14 | 48 | 2.84E-20 | 35 | 6.27E-15 |
| FAM63A | 34 | 6.20E-14 | 36 | 6.32E-21 | 35 | 3.10E-14 |
| PINK1 | 37 | 9.12E-14 | 33 | 4.33E-21 | 35 | 4.56E-14 |
| ARHGAP10 | 65 | 1.23E-12 | 9 | 1.33E-24 | 37 | 6.13E-13 |
| ACOX3 | 36 | 7.86E-14 | 39 | 9.66E-21 | 37.5 | 3.93E-14 |
| GDPD3 | 5 | 1.48E-16 | 77 | 4.99E-19 | 41 | 7.43E-17 |
| SH3GLB2 | 1 | 3.77E-18 | 85 | 7.12E-19 | 43 | 2.24E-18 |
| FUT6 | 67 | 1.29E-12 | 25 | 3.96E-22 | 46 | 6.47E-13 |
| KIAA0232 | 62 | 1.12E-12 | 37 | 8.98E-21 | 49.5 | 5.59E-13 |
| EMP1 | 13 | 4.44E-15 | 92 | 9.82E-19 | 52.5 | 2.22E-15 |
| LRRFIP2 | 74 | 2.63E-12 | 32 | 3.93E-21 | 53 | 1.32E-12 |
| KLK13 | 52 | 7.06E-13 | 56 | 5.72E-20 | 54 | 3.53E-13 |
| VPS13D | 60 | 9.93E-13 | 69 | 3.86E-19 | 64.5 | 4.96E-13 |
| SASH1 | 113 | 1.54E-11 | 16 | 2.96E-23 | 64.5 | 7.69E-12 |
| IL17RC | 129 | 3.11E-11 | 3 | 3.58E-25 | 66 | 1.56E-11 |
| CEACAM1 | 97 | 6.65E-12 | 47 | 2.68E-20 | 72 | 3.33E-12 |
| EPS8L1 | 45 | 3.42E-13 | 108 | 2.67E-18 | 76.5 | 1.71E-13 |
| ABLIM3 | 43 | 2.33E-13 | 112 | 2.90E-18 | 77.5 | 1.17E-13 |
| C2orf24 | 33 | 5.29E-14 | 123 | 7.23E-18 | 78 | 2.64E-14 |
| EHD3 | 32 | 5.27E-14 | 125 | 1.08E-17 | 78.5 | 2.63E-14 |
| CPEB3 | 41 | 1.84E-13 | 116 | 4.70E-18 | 78.5 | 9.19E-14 |
| KLF8 | 106 | 1.36E-11 | 52 | 4.13E-20 | 79 | 6.82E-12 |
| C1orf116 | 27 | 2.94E-14 | 134 | 1.62E-17 | 80.5 | 1.47E-14 |
| OBFC1 | 64 | 1.19E-12 | 97 | 1.49E-18 | 80.5 | 5.93E-13 |
| CYP2J2 | 55 | 7.98E-13 | 107 | 2.47E-18 | 81 | 3.99E-13 |
| LMBRD1 | 96 | 6.61E-12 | 68 | 3.71E-19 | 82 | 3.31E-12 |
| FUT3 | 90 | 5.40E-12 | 78 | 5.14E-19 | 84 | 2.70E-12 |
| EPB41L3 | 8 | 5.84E-16 | 161 | 8.23E-17 | 84.5 | 3.33E-16 |
| EPB41L4A | 54 | 7.66E-13 | 117 | 4.71E-18 | 85.5 | 3.83E-13 |
| ACAA1 | 10 | 2.75E-15 | 164 | 9.15E-17 | 87 | 1.42E-15 |
| CXCR2 | 42 | 2.04E-13 | 133 | 1.55E-17 | 87.5 | 1.02E-13 |
| GPD1L | 170 | 1.20E-10 | 5 | 8.15E-25 | 87.5 | 6.00E-11 |
| GAB2 | 86 | 5.00E-12 | 90 | 8.98E-19 | 88 | 2.50E-12 |
| C2orf54 | 21 | 8.34E-15 | 157 | 7.54E-17 | 89 | 4.21E-15 |
| SCNN1B | 61 | 1.08E-12 | 119 | 5.70E-18 | 90 | 5.40E-13 |
| HPGD | 172 | 1.27E-10 | 12 | 1.40E-23 | 92 | 6.37E-11 |
| EPS8L2 | 91 | 5.40E-12 | 102 | 1.77E-18 | 96.5 | 2.70E-12 |
| PDCD4 | 59 | 9.86E-13 | 141 | 2.52E-17 | 100 | 4.93E-13 |
| ABLIM1 | 87 | 5.07E-12 | 115 | 4.41E-18 | 101 | 2.54E-12 |
| GAB1 | 123 | 2.40E-11 | 81 | 6.11E-19 | 102 | 1.20E-11 |
| MGLL | 179 | 2.10E-10 | 35 | 4.94E-21 | 107 | 1.05E-10 |
| TRIP10 | 158 | 6.53E-11 | 67 | 3.53E-19 | 112.5 | 3.26E-11 |
| TPCN1 | 207 | 5.10E-10 | 18 | 5.61E-23 | 112.5 | 2.55E-10 |
| SLC16A6 | 12 | 3.76E-15 | 214 | 7.15E-16 | 113 | 2.24E-15 |
| SLURP1 | 93 | 5.55E-12 | 137 | 1.85E-17 | 115 | 2.77E-12 |
| ARHGEF10L | 196 | 3.56E-10 | 34 | 4.54E-21 | 115 | 1.78E-10 |
| RANBP9 | 16 | 5.19E-15 | 228 | 1.01E-15 | 122 | 3.10E-15 |
| PRSS3 | 68 | 1.62E-12 | 178 | 1.63E-16 | 123 | 8.08E-13 |
| TJP1 | 78 | 3.50E-12 | 170 | 1.32E-16 | 124 | 1.75E-12 |
| SLC37A1 | 168 | 1.01E-10 | 80 | 6.10E-19 | 124 | 5.04E-11 |
| BLNK | 80 | 3.98E-12 | 184 | 2.15E-16 | 132 | 1.99E-12 |
| CWH43 | 4 | 1.47E-16 | 262 | 2.15E-15 | 133 | 1.15E-15 |
| ANO10 | 227 | 9.05E-10 | 42 | 1.31E-20 | 134.5 | 4.53E-10 |
| COBL | 151 | 5.71E-11 | 121 | 6.38E-18 | 136 | 2.86E-11 |
| HIGD1A | 181 | 2.17E-10 | 96 | 1.46E-18 | 138.5 | 1.09E-10 |
| TRIOBP | 94 | 5.82E-12 | 187 | 2.24E-16 | 140.5 | 2.91E-12 |
| ANXA11 | 120 | 2.18E-11 | 165 | 1.02E-16 | 142.5 | 1.09E-11 |
| RAB5B | 154 | 5.89E-11 | 132 | 1.53E-17 | 143 | 2.95E-11 |
| NCOA1 | 211 | 5.38E-10 | 76 | 4.89E-19 | 143.5 | 2.69E-10 |
| IL1F6 | 148 | 4.92E-11 | 140 | 2.42E-17 | 144 | 2.46E-11 |
| SH3GL1 | 57 | 8.38E-13 | 234 | 1.17E-15 | 145.5 | 4.20E-13 |
| SLC24A3 | 51 | 6.20E-13 | 246 | 1.55E-15 | 148.5 | 3.11E-13 |
| TMPRSS2 | 184 | 2.47E-10 | 114 | 3.02E-18 | 149 | 1.23E-10 |
| DLG2 | 63 | 1.16E-12 | 237 | 1.29E-15 | 150 | 5.83E-13 |
| TTC9 | 122 | 2.34E-11 | 180 | 1.68E-16 | 151 | 1.17E-11 |
| ACOX1 | 81 | 4.17E-12 | 226 | 9.68E-16 | 153.5 | 2.09E-12 |
| CCNG2 | 126 | 2.86E-11 | 181 | 1.79E-16 | 153.5 | 1.43E-11 |
| ECM1 | 136 | 3.82E-11 | 173 | 1.42E-16 | 154.5 | 1.91E-11 |
| ABHD5 | 101 | 8.17E-12 | 209 | 5.96E-16 | 155 | 4.09E-12 |
| VAT1 | 25 | 1.95E-14 | 297 | 4.65E-15 | 161 | 1.21E-14 |
| SECISBP2L | 189 | 2.98E-10 | 136 | 1.74E-17 | 162.5 | 1.49E-10 |
| ALS2CL | 75 | 2.96E-12 | 253 | 1.73E-15 | 164 | 1.48E-12 |
| PRSS2 | 49 | 4.39E-13 | 280 | 3.22E-15 | 164.5 | 2.21E-13 |
| TOLLIP | 70 | 2.13E-12 | 260 | 2.08E-15 | 165 | 1.07E-12 |
| ITPR2 | 39 | 1.39E-13 | 292 | 4.11E-15 | 165.5 | 7.17E-14 |
| RNF141 | 11 | 3.13E-15 | 321 | 1.02E-14 | 166 | 6.68E-15 |
| RNF10 | 267 | 1.94E-09 | 73 | 4.52E-19 | 170 | 9.68E-10 |
| PHACTR2 | 190 | 3.00E-10 | 154 | 5.28E-17 | 172 | 1.50E-10 |
| SCAMP2 | 146 | 4.61E-11 | 200 | 4.71E-16 | 173 | 2.31E-11 |
| ROR1 | 274 | 2.21E-09 | 79 | 5.85E-19 | 176.5 | 1.10E-09 |
| TMOD3 | 73 | 2.43E-12 | 282 | 3.67E-15 | 177.5 | 1.22E-12 |
| NDRG2 | 347 | 7.48E-09 | 10 | 5.19E-24 | 178.5 | 3.74E-09 |
| RORA | 142 | 4.28E-11 | 219 | 8.03E-16 | 180.5 | 2.14E-11 |
| TP53I3 | 92 | 5.50E-12 | 270 | 2.59E-15 | 181 | 2.75E-12 |
| STK39 | 232 | 9.66E-10 | 131 | 1.46E-17 | 181.5 | 4.83E-10 |
| GALNT12 | 299 | 3.44E-09 | 64 | 1.58E-19 | 181.5 | 1.72E-09 |
| MSRA | 324 | 5.19E-09 | 41 | 1.24E-20 | 182.5 | 2.59E-09 |
| CLIC3 | 194 | 3.43E-10 | 176 | 1.50E-16 | 185 | 1.72E-10 |
| CCDC6 | 112 | 1.52E-11 | 261 | 2.13E-15 | 186.5 | 7.62E-12 |
| TTC39A | 175 | 1.60E-10 | 198 | 4.32E-16 | 186.5 | 8.00E-11 |
| HEBP2 | 69 | 2.04E-12 | 306 | 5.68E-15 | 187.5 | 1.02E-12 |
| SNX24 | 28 | 3.26E-14 | 355 | 2.88E-14 | 191.5 | 3.07E-14 |
| PMM1 | 130 | 3.22E-11 | 255 | 1.83E-15 | 192.5 | 1.61E-11 |
| CD24 | 222 | 8.17E-10 | 167 | 1.13E-16 | 194.5 | 4.08E-10 |
| SCEL | 266 | 1.90E-09 | 130 | 1.37E-17 | 198 | 9.49E-10 |
| UNC13B | 288 | 3.02E-09 | 113 | 2.96E-18 | 200.5 | 1.51E-09 |
| PADI1 | 336 | 5.97E-09 | 65 | 2.33E-19 | 200.5 | 2.98E-09 |
| MAPK3 | 246 | 1.46E-09 | 162 | 8.29E-17 | 204 | 7.32E-10 |
| ALDH9A1 | 328 | 5.35E-09 | 83 | 6.51E-19 | 205.5 | 2.68E-09 |
| BRP44L | 284 | 2.87E-09 | 129 | 1.30E-17 | 206.5 | 1.44E-09 |
| DOK4 | 66 | 1.27E-12 | 353 | 2.78E-14 | 209.5 | 6.47E-13 |
| ZNF365 | 251 | 1.53E-09 | 168 | 1.14E-16 | 209.5 | 7.66E-10 |
| KAZ | 183 | 2.45E-10 | 240 | 1.37E-15 | 211.5 | 1.23E-10 |
| WWC1 | 82 | 4.24E-12 | 342 | 2.18E-14 | 212 | 2.13E-12 |
| ID4 | 279 | 2.60E-09 | 145 | 3.39E-17 | 212 | 1.30E-09 |
| CHP | 44 | 2.72E-13 | 382 | 6.67E-14 | 213 | 1.69E-13 |
| NAGK | 71 | 2.24E-12 | 356 | 2.92E-14 | 213.5 | 1.13E-12 |
| RASAL1 | 128 | 3.09E-11 | 301 | 5.26E-15 | 214.5 | 1.55E-11 |
| SPINK5 | 258 | 1.79E-09 | 172 | 1.36E-16 | 215 | 8.95E-10 |
| ACADVL | 262 | 1.87E-09 | 169 | 1.18E-16 | 215.5 | 9.34E-10 |
| SYNGR1 | 373 | 9.84E-09 | 59 | 6.04E-20 | 216 | 4.92E-09 |
| AGFG2 | 350 | 7.73E-09 | 84 | 6.63E-19 | 217 | 3.86E-09 |
| SLC35C1 | 160 | 6.96E-11 | 275 | 2.86E-15 | 217.5 | 3.48E-11 |
| TMEM111 | 223 | 8.78E-10 | 213 | 7.13E-16 | 218 | 4.39E-10 |
| MAPKAPK3 | 226 | 9.04E-10 | 216 | 7.41E-16 | 221 | 4.52E-10 |
| CHMP2B | 237 | 1.07E-09 | 205 | 5.25E-16 | 221 | 5.35E-10 |
| GAS7 | 376 | 1.07E-08 | 66 | 3.20E-19 | 221 | 5.37E-09 |
| CAPN5 | 400 | 1.59E-08 | 43 | 1.43E-20 | 221.5 | 7.96E-09 |
| FNDC4 | 177 | 1.78E-10 | 272 | 2.79E-15 | 224.5 | 8.89E-11 |
| SLC16A7 | 23 | 1.28E-14 | 427 | 2.12E-13 | 225 | 1.12E-13 |
| SLC13A4 | 187 | 2.54E-10 | 265 | 2.23E-15 | 226 | 1.27E-10 |
| WDR26 | 228 | 9.10E-10 | 225 | 9.52E-16 | 226.5 | 4.55E-10 |
| MXD1 | 138 | 3.86E-11 | 316 | 7.30E-15 | 227 | 1.93E-11 |
| TRIM13 | 164 | 9.45E-11 | 293 | 4.40E-15 | 228.5 | 4.72E-11 |
| TGM3 | 358 | 8.68E-09 | 99 | 1.54E-18 | 228.5 | 4.34E-09 |
| CPPED1 | 192 | 3.22E-10 | 266 | 2.31E-15 | 229 | 1.61E-10 |
| MAPT | 313 | 4.24E-09 | 146 | 3.51E-17 | 229.5 | 2.12E-09 |
| EVPL | 118 | 1.90E-11 | 343 | 2.18E-14 | 230.5 | 9.53E-12 |
| NPEPPS | 100 | 8.10E-12 | 363 | 3.59E-14 | 231.5 | 4.07E-12 |
| MTERFD2 | 161 | 7.57E-11 | 303 | 5.46E-15 | 232 | 3.79E-11 |
| ELMO2 | 178 | 1.86E-10 | 286 | 3.88E-15 | 232 | 9.28E-11 |
| SULT2B1 | 210 | 5.33E-10 | 256 | 1.83E-15 | 233 | 2.67E-10 |
| DUSP5 | 200 | 3.85E-10 | 268 | 2.36E-15 | 234 | 1.92E-10 |
| FLG2 | 166 | 9.98E-11 | 308 | 5.81E-15 | 237 | 4.99E-11 |
| RARG | 193 | 3.43E-10 | 281 | 3.65E-15 | 237 | 1.71E-10 |
| TAX1BP1 | 205 | 4.74E-10 | 269 | 2.52E-15 | 237 | 2.37E-10 |
| FLG | 167 | 9.98E-11 | 309 | 5.81E-15 | 238 | 4.99E-11 |
| LRP10 | 234 | 1.04E-09 | 243 | 1.46E-15 | 238.5 | 5.22E-10 |
| GYS2 | 114 | 1.59E-11 | 367 | 4.02E-14 | 240.5 | 7.99E-12 |
| MAL | 388 | 1.42E-08 | 94 | 1.04E-18 | 241 | 7.09E-09 |
| DHRS12 | 365 | 9.06E-09 | 126 | 1.09E-17 | 245.5 | 4.53E-09 |
| CES2 | 308 | 3.79E-09 | 185 | 2.20E-16 | 246.5 | 1.90E-09 |
| TFAP2B | 18 | 7.40E-15 | 477 | 6.97E-13 | 247.5 | 3.52E-13 |
| LOC441453 | 15 | 5.12E-15 | 482 | 8.67E-13 | 248.5 | 4.36E-13 |
| LPIN1 | 469 | 4.25E-08 | 29 | 7.21E-22 | 249 | 2.13E-08 |
| CDKN2AIP | 88 | 5.26E-12 | 415 | 1.62E-13 | 251.5 | 2.71E-12 |
| AHNAK | 326 | 5.23E-09 | 179 | 1.65E-16 | 252.5 | 2.62E-09 |
| PHACTR4 | 355 | 8.51E-09 | 150 | 4.23E-17 | 252.5 | 4.25E-09 |
| CAST | 150 | 5.29E-11 | 361 | 3.35E-14 | 255.5 | 2.65E-11 |
| CITED2 | 119 | 1.97E-11 | 400 | 9.86E-14 | 259.5 | 9.89E-12 |
| MYO6 | 171 | 1.20E-10 | 348 | 2.49E-14 | 259.5 | 6.01E-11 |
| ACPP | 89 | 5.28E-12 | 432 | 2.28E-13 | 260.5 | 2.76E-12 |
| SORBS2 | 498 | 5.84E-08 | 26 | 4.10E-22 | 262 | 2.92E-08 |
| WNK1 | 53 | 7.45E-13 | 472 | 6.17E-13 | 262.5 | 6.81E-13 |
| KCNAB1 | 466 | 4.18E-08 | 62 | 1.20E-19 | 264 | 2.09E-08 |
| DOPEY2 | 218 | 6.79E-10 | 311 | 5.97E-15 | 264.5 | 3.39E-10 |
| SIM2 | 516 | 7.02E-08 | 13 | 1.45E-23 | 264.5 | 3.51E-08 |
| SERPINB13 | 76 | 3.37E-12 | 456 | 4.13E-13 | 266 | 1.89E-12 |
| C18orf25 | 243 | 1.30E-09 | 289 | 3.95E-15 | 266 | 6.49E-10 |
| NAPA | 162 | 8.04E-11 | 373 | 4.33E-14 | 267.5 | 4.02E-11 |
| BBOX1 | 14 | 4.99E-15 | 525 | 2.57E-12 | 269.5 | 1.29E-12 |
| PLEKHG6 | 104 | 1.11E-11 | 441 | 3.20E-13 | 272.5 | 5.69E-12 |
| EXPH5 | 155 | 5.91E-11 | 390 | 7.76E-14 | 272.5 | 2.96E-11 |
| GPX3 | 490 | 5.53E-08 | 58 | 5.97E-20 | 274 | 2.77E-08 |
| CRNN | 335 | 5.90E-09 | 215 | 7.32E-16 | 275 | 2.95E-09 |
| GMDS | 117 | 1.90E-11 | 439 | 2.95E-13 | 278 | 9.66E-12 |
| SLK | 115 | 1.65E-11 | 443 | 3.26E-13 | 279 | 8.39E-12 |
| ASAP3 | 471 | 4.38E-08 | 89 | 8.22E-19 | 280 | 2.19E-08 |
| DHRS1 | 77 | 3.42E-12 | 484 | 8.77E-13 | 280.5 | 2.15E-12 |
| PDLIM5 | 173 | 1.50E-10 | 393 | 8.14E-14 | 283 | 7.50E-11 |
| CYP4B1 | 254 | 1.64E-09 | 318 | 8.68E-15 | 286 | 8.19E-10 |
| HOPX | 256 | 1.75E-09 | 319 | 9.88E-15 | 287.5 | 8.76E-10 |
| SLC25A23 | 282 | 2.72E-09 | 294 | 4.49E-15 | 288 | 1.36E-09 |
| KANK1 | 369 | 9.40E-09 | 208 | 5.85E-16 | 288.5 | 4.70E-09 |
| UPK1A | 268 | 1.96E-09 | 312 | 6.01E-15 | 290 | 9.79E-10 |
| ADH1B | 520 | 7.24E-08 | 61 | 6.30E-20 | 290.5 | 3.62E-08 |
| KLF4 | 311 | 3.88E-09 | 277 | 3.15E-15 | 294 | 1.94E-09 |
| FMO2 | 513 | 6.88E-08 | 88 | 7.53E-19 | 300.5 | 3.44E-08 |
| CYP2C18 | 263 | 1.87E-09 | 339 | 1.96E-14 | 301 | 9.36E-10 |
| CAB39L | 587 | 1.51E-07 | 15 | 1.59E-23 | 301 | 7.55E-08 |
| ZNF426 | 393 | 1.49E-08 | 210 | 6.01E-16 | 301.5 | 7.43E-09 |
| CLCA4 | 480 | 5.08E-08 | 124 | 9.30E-18 | 302 | 2.54E-08 |
| PDLIM2 | 357 | 8.62E-09 | 251 | 1.69E-15 | 304 | 4.31E-09 |
| CRCT1 | 318 | 4.70E-09 | 291 | 4.10E-15 | 304.5 | 2.35E-09 |
| PLEKHM1 | 325 | 5.20E-09 | 284 | 3.80E-15 | 304.5 | 2.60E-09 |
| BLOC1S1 | 252 | 1.55E-09 | 360 | 3.31E-14 | 306 | 7.76E-10 |
| NLRX1 | 208 | 5.13E-10 | 407 | 1.18E-13 | 307.5 | 2.57E-10 |
| LOC100287917 | 278 | 2.60E-09 | 337 | 1.86E-14 | 307.5 | 1.30E-09 |
| RAB11A | 109 | 1.46E-11 | 507 | 1.52E-12 | 308 | 8.04E-12 |
