# Supplementary material for: Microarray analyses reveal genes related to progression and prognosis of esophageal squamous cell carcinoma
Source: Oncotarget. 2017 Aug 12;8(45):78838–50. doi: 10.18632/oncotarget.20232 (PMC5668002; doi:10.18632/oncotarget.20232)
Supplement: Supplementary file 4 [file oncotarget-08-78838-s004.docx]

**Supplementary Table 3: Relationships between of candidate genes**

| Gene 1 | Gene 2 | Weight | Type | Source |
| --- | --- | --- | --- | --- |
| AEN | PES1 | 0.082312431 | Co-expression | Boldrick-Relman-2002 |
| MGLL_HUMAN | TFRC | 0.041402786 | Co-expression | Boldrick-Relman-2002 |
| RRM2 | TFRC | 0.05894246 | Co-expression | Boldrick-Relman-2002 |
| S100A10 | TP53I3 | 0.043692142 | Co-expression | Boldrick-Relman-2002 |
| CENPF | RRM2 | 0.170124968 | Co-expression | Chen-Brown-2002 |
| ITGA6 | TP53I3 | 0.329332528 | Co-expression | Chen-Brown-2002 |
| PAXIP1 | TOPBP1 | 0.11991171 | Co-expression | Chen-Brown-2002 |
| RRM2 | TOPBP1 | 0.091939783 | Co-expression | Chen-Brown-2002 |
| S100A10 | ITGA6 | 0.250263063 | Co-expression | Chen-Brown-2002 |
| S100A10 | TFRC | 0.218153999 | Co-expression | Chen-Brown-2002 |
| S100A10 | TP53I3 | 0.24467754 | Co-expression | Chen-Brown-2002 |
| TAX1BP3 | TRIP10 | 0.113360871 | Co-expression | Chen-Brown-2002 |
| UBE2E3 | TOPBP1 | 0.106748912 | Co-expression | Chen-Brown-2002 |
| LGALS1 | MGLL_HUMAN | 0.333176362 | Co-expression | Cheok-Evans-2003 |
| LGALS1 | TPM4 | 0.284438985 | Co-expression | Cheok-Evans-2003 |
| MGLL_HUMAN | TFRC | 0.327704702 | Co-expression | Cheok-Evans-2003 |
| PAXIP1 | ABHD14A | 0.109923786 | Co-expression | Cheok-Evans-2003 |
| TPM4 | MGLL_HUMAN | 0.286607774 | Co-expression | Cheok-Evans-2003 |
| TPM4 | TFRC | 0.297848225 | Co-expression | Cheok-Evans-2003 |
| UBE2E3 | MGLL_HUMAN | 0.354243139 | Co-expression | Cheok-Evans-2003 |
| UBE2E3 | TFRC | 0.38832329 | Co-expression | Cheok-Evans-2003 |
| AEN | NETO2_HUMAN | 0.406330116 | Co-expression | Gysin-McMahon-2012 |
| CENPF | DBF4 | 0.125971535 | Co-expression | Gysin-McMahon-2012 |
| CENPF | ECT2_HUMAN | 0.253983152 | Co-expression | Gysin-McMahon-2012 |
| CENPF | PAXIP1 | 0.216778246 | Co-expression | Gysin-McMahon-2012 |
| CENPF | STIL | 0.264141553 | Co-expression | Gysin-McMahon-2012 |
| CENPF | TOPBP1 | 0.263264056 | Co-expression | Gysin-McMahon-2012 |
| DBF4 | ECT2_HUMAN | 0.089621717 | Co-expression | Gysin-McMahon-2012 |
| DBF4 | RRM2 | 0.096380839 | Co-expression | Gysin-McMahon-2012 |
| DBF4 | TOPBP1 | 0.083279262 | Co-expression | Gysin-McMahon-2012 |
| RRM2 | ECT2_HUMAN | 0.201362647 | Co-expression | Gysin-McMahon-2012 |
| RRM2 | TFRC | 0.29525221 | Co-expression | Gysin-McMahon-2012 |
| RRM2 | TOPBP1 | 0.220796182 | Co-expression | Gysin-McMahon-2012 |
| SMC4 | DBF4 | 0.134516605 | Co-expression | Gysin-McMahon-2012 |
| SMC4 | ECT2_HUMAN | 0.327748071 | Co-expression | Gysin-McMahon-2012 |
| STIL | DBF4 | 0.085330169 | Co-expression | Gysin-McMahon-2012 |
| STIL | ECT2_HUMAN | 0.176897801 | Co-expression | Gysin-McMahon-2012 |
| STIL | RRM2 | 0.21608158 | Co-expression | Gysin-McMahon-2012 |
| STIL | TFRC | 0.2087414 | Co-expression | Gysin-McMahon-2012 |
| STIL | TOPBP1 | 0.205725787 | Co-expression | Gysin-McMahon-2012 |
| TAX1BP3 | TP53I3 | 0.248065882 | Co-expression | Gysin-McMahon-2012 |
| TOPBP1 | ECT2_HUMAN | 0.199663019 | Co-expression | Gysin-McMahon-2012 |
| TOPBP1 | TFRC | 0.219002706 | Co-expression | Gysin-McMahon-2012 |
| PTDSS1 | TFRC | 0.09708079 | Co-expression | Mallon-McKay-2013 |
| RRM2 | NETO2_HUMAN | 0.212299182 | Co-expression | Mallon-McKay-2013 |
| SMC4 | NETO2_HUMAN | 0.094096973 | Co-expression | Mallon-McKay-2013 |
| STIL | NETO2_HUMAN | 0.151339984 | Co-expression | Mallon-McKay-2013 |
| STIL | PAXIP1 | 0.072443177 | Co-expression | Mallon-McKay-2013 |
| STIL | RRM2 | 0.173571447 | Co-expression | Mallon-McKay-2013 |
| STIL | SMC4 | 0.076950107 | Co-expression | Mallon-McKay-2013 |
| LGALS1 | S100A10 | 0.119613343 | Co-expression | Ramaswamy-Golub-2001 |
| MPHOSPH6 | ITGA6 | 0.093261173 | Co-expression | Ramaswamy-Golub-2001 |
| MPHOSPH6 | PTDSS1 | 0.060634691 | Co-expression | Ramaswamy-Golub-2001 |
| TAX1BP3 | TRIP10 | 0.032256591 | Co-expression | Ramaswamy-Golub-2001 |
| TOPBP1 | PTDSS1 | 0.056814212 | Co-expression | Ramaswamy-Golub-2001 |
| AEN | NETO2_HUMAN | 0.241317411 | Co-expression | Salaverria-Siebert-2011 |
| AEN | PES1 | 0.099851371 | Co-expression | Salaverria-Siebert-2011 |
| AEN | TFRC | 0.262114145 | Co-expression | Salaverria-Siebert-2011 |
| CENPF | ECT2_HUMAN | 0.233252891 | Co-expression | Salaverria-Siebert-2011 |
| CENPF | STIL | 0.182186809 | Co-expression | Salaverria-Siebert-2011 |
| DBF4 | ECT2_HUMAN | 0.091381954 | Co-expression | Salaverria-Siebert-2011 |
| DBF4 | PAXIP1 | 0.101281962 | Co-expression | Salaverria-Siebert-2011 |
| DBF4 | TOPBP1 | 0.063869527 | Co-expression | Salaverria-Siebert-2011 |
| LGALS1 | TAX1BP3 | 0.311455949 | Co-expression | Salaverria-Siebert-2011 |
| LGALS1 | TP53I3 | 0.398821577 | Co-expression | Salaverria-Siebert-2011 |
| LGALS1 | TRIP10 | 0.332205394 | Co-expression | Salaverria-Siebert-2011 |
| MPHOSPH6 | AEN | 0.233353086 | Co-expression | Salaverria-Siebert-2011 |
| MPHOSPH6 | NETO2_HUMAN | 0.338878053 | Co-expression | Salaverria-Siebert-2011 |
| MPHOSPH6 | TFRC | 0.334827385 | Co-expression | Salaverria-Siebert-2011 |
| PAXIP1 | TOPBP1 | 0.123555184 | Co-expression | Salaverria-Siebert-2011 |
| PES1 | TFRC | 0.135215263 | Co-expression | Salaverria-Siebert-2011 |
| RRM2 | ECT2_HUMAN | 0.182586104 | Co-expression | Salaverria-Siebert-2011 |
| S100A10 | TP53I3 | 0.435602814 | Co-expression | Salaverria-Siebert-2011 |
| S100A10 | TRIP10 | 0.379633718 | Co-expression | Salaverria-Siebert-2011 |
| SMC4 | ECT2_HUMAN | 0.316434763 | Co-expression | Salaverria-Siebert-2011 |
| SMC4 | TOPBP1 | 0.22228845 | Co-expression | Salaverria-Siebert-2011 |
| STIL | ECT2_HUMAN | 0.111147563 | Co-expression | Salaverria-Siebert-2011 |
| STIL | RRM2 | 0.140363445 | Co-expression | Salaverria-Siebert-2011 |
| STIL | SMC4 | 0.199910865 | Co-expression | Salaverria-Siebert-2011 |
| TAX1BP3 | TP53I3 | 0.392439519 | Co-expression | Salaverria-Siebert-2011 |
| TAX1BP3 | TRIP10 | 0.347456865 | Co-expression | Salaverria-Siebert-2011 |
| TFRC | NETO2_HUMAN | 0.365242425 | Co-expression | Salaverria-Siebert-2011 |
| TOPBP1 | ECT2_HUMAN | 0.119112751 | Co-expression | Salaverria-Siebert-2011 |
| TPM4 | TP53I3 | 0.411223669 | Co-expression | Salaverria-Siebert-2011 |
| UBE2E3 | NETO2_HUMAN | 0.195930656 | Co-expression | Salaverria-Siebert-2011 |
| CENPF | ECT2_HUMAN | 0.015647121 | Co-expression | Smirnov-Cheung-2009 |
| DBF4 | ECT2_HUMAN | 0.008795598 | Co-expression | Smirnov-Cheung-2009 |
| DBF4 | PAXIP1 | 0.007255058 | Co-expression | Smirnov-Cheung-2009 |
| DBF4 | TOPBP1 | 0.010487811 | Co-expression | Smirnov-Cheung-2009 |
| LGALS1 | MGLL_HUMAN | 0.010750814 | Co-expression | Smirnov-Cheung-2009 |
| LGALS1 | PTDSS1 | 0.024671334 | Co-expression | Smirnov-Cheung-2009 |
| MGLL_HUMAN | NETO2_HUMAN | 0.008873979 | Co-expression | Smirnov-Cheung-2009 |
| MPHOSPH6 | TFRC | 0.026654294 | Co-expression | Smirnov-Cheung-2009 |
| PAXIP1 | TOPBP1 | 0.013013851 | Co-expression | Smirnov-Cheung-2009 |
| RRM2 | ITGA6 | 0.018617975 | Co-expression | Smirnov-Cheung-2009 |
| RRM2 | S100A10 | 0.026122233 | Co-expression | Smirnov-Cheung-2009 |
| SMC4 | DBF4 | 0.016439682 | Co-expression | Smirnov-Cheung-2009 |
| SMC4 | ECT2_HUMAN | 0.017751262 | Co-expression | Smirnov-Cheung-2009 |
| SMC4 | TOPBP1 | 0.027157104 | Co-expression | Smirnov-Cheung-2009 |
| STIL | ECT2_HUMAN | 0.011538187 | Co-expression | Smirnov-Cheung-2009 |
| TAX1BP3 | TP53I3 | 0.005056684 | Co-expression | Smirnov-Cheung-2009 |
| CENPF | DBF4 | 0.041662622 | Co-localization | Johnson-Shoemaker-2003 |
| CENPF | SMC4 | 0.119480303 | Co-localization | Johnson-Shoemaker-2003 |
| CENPF | TOPBP1 | 0.074430393 | Co-localization | Johnson-Shoemaker-2003 |
| DBF4 | TOPBP1 | 0.021880376 | Co-localization | Johnson-Shoemaker-2003 |
| ITGA6 | TP53I3 | 0.065007648 | Co-localization | Johnson-Shoemaker-2003 |
| LGALS1 | TAX1BP3 | 0.061127153 | Co-localization | Johnson-Shoemaker-2003 |
| S100A10 | TP53I3 | 0.075069557 | Co-localization | Johnson-Shoemaker-2003 |
| STIL | DBF4 | 0.01905756 | Co-localization | Johnson-Shoemaker-2003 |
| STIL | TFRC | 0.0465042 | Co-localization | Johnson-Shoemaker-2003 |
| TAX1BP3 | S100A10 | 0.081345702 | Co-localization | Johnson-Shoemaker-2003 |
| TAX1BP3 | TP53I3 | 0.132961021 | Co-localization | Johnson-Shoemaker-2003 |
| ABHD14A | MGLL_HUMAN | 1.732395016 | Shared protein domains | PFAM |
| DAGLB | MGLL_HUMAN | 0.91061675 | Shared protein domains | PFAM |
| DBF4 | ECT2_HUMAN | 0.299091105 | Shared protein domains | PFAM |
| DBF4 | MCPH1 | 0.550384038 | Shared protein domains | PFAM |
| DBF4 | PAXIP1 | 0.550385681 | Shared protein domains | PFAM |
| DBF4 | PES1 | 0.550384038 | Shared protein domains | PFAM |
| DBF4 | TOPBP1 | 0.550385681 | Shared protein domains | PFAM |
| MCPH1 | ECT2_HUMAN | 0.392748015 | Shared protein domains | PFAM |
| MCPH1 | PAXIP1 | 0.72273258 | Shared protein domains | PFAM |
| MCPH1 | TOPBP1 | 0.72273258 | Shared protein domains | PFAM |
| NAALADL1 | TFR2 | 0.849406819 | Shared protein domains | PFAM |
| NAALADL1 | TFRC | 0.849406819 | Shared protein domains | PFAM |
| PAXIP1 | ECT2_HUMAN | 0.392749213 | Shared protein domains | PFAM |
| PAXIP1 | TOPBP1 | 0.722734771 | Shared protein domains | PFAM |
| PES1 | ECT2_HUMAN | 0.392748015 | Shared protein domains | PFAM |
| PES1 | MCPH1 | 0.722730253 | Shared protein domains | PFAM |
| PES1 | PAXIP1 | 0.72273258 | Shared protein domains | PFAM |
| PES1 | TOPBP1 | 0.72273258 | Shared protein domains | PFAM |
| PTDSS2 | PTDSS1 | 9.188686892 | Shared protein domains | PFAM |
| TFR2 | TFRC | 0.849406819 | Shared protein domains | PFAM |
| TOPBP1 | ECT2_HUMAN | 0.392749213 | Shared protein domains | PFAM |
